# Multilevel Characteristics of Cumulative Symptom Burden in Young Survivors of Childhood Cancer

**DOI:** 10.1001/jamanetworkopen.2024.10145

**Published:** 2024-05-07

**Authors:** Madeline R. Horan, Deo Kumar Srivastava, Jaesung Choi, Kevin R. Krull, Gregory T. Armstrong, Kirsten K. Ness, Melissa M. Hudson, Justin N. Baker, I-Chan Huang

**Affiliations:** 1 Department of Epidemiology and Cancer Control, St Jude Children’s Research Hospital, Memphis, Tennessee; 2 Department of Biostatistics, St Jude Children’s Research Hospital, Memphis, Tennessee; 3 Department of Psychology and Biobehavioral Sciences, St Jude Children’s Research Hospital, Memphis, Tennessee; 4 Department of Oncology, St Jude Children's Research Hospital, Memphis, Tennessee; 5 Department of Pediatrics, Stanford University School of Medicine, Stanford, California

## Abstract

**Importance:**

Symptom burden and its characteristics among survivors of pediatric cancers aged 8 to 18 years remain understudied.

**Objective:**

To examine the prevalence of symptom burden among young childhood cancer survivors and identify associations with sociodemographic, clinical, and psychological resilience skills, and health-related quality of life (HRQOL).

**Design, Setting, and Participants:**

A cross-sectional analysis using data collected from November 1, 2017, to January 31, 2019, in a survivorship clinic at a US-based comprehensive cancer center was conducted. Participants included 302 dyads of children aged 8 to 18 years who survived at least 5 years beyond diagnosis and their primary caregivers. Data analysis was performed from March 13, 2023, to February 29, 2024.

**Exposures:**

Diagnosis, caregiver-reported family conflict, self-reported caregiver anxiety, neighborhood-level social vulnerability, and survivor-reported meaning and purpose.

**Main Outcomes and Measures:**

Novel symptom-level burden, integrating the attributes of severity and daily activity interference using the pediatric version of the Patient-Reported Outcomes Version of the Common Terminology Criteria for Adverse Events, global cumulative symptom burden, and HRQOL using the EuroQol-5D. Multinomial logistic regression identified characteristics associated with symptom burden; linear regression assessed symptom burden and HRQOL associations.

**Results:**

Among 302 survivors (mean [SD] age, 14.2 [2.9] years, mean [SD] time since diagnosis, 10.9 [2.9] years; 153 [50.7%] male), 186 (62.0%) had low, 77 (25.7%) moderate, and 37 (12.3%) high global cumulative symptom burden. Greater caregiver anxiety was associated with moderate (risk ratio [RR], 1.56; 95% CI, 1.09-2.24) global symptom burden. Greater neighborhood deprivation was associated with moderate global symptom burden (RR, 4.86; 95% CI, 1.29-18.26). Survivors with greater meaning/purpose were less likely to have moderate (RR, 0.42; 95% CI, 0.29-0.61) and high (RR, 0.27; 95% CI, 0.16-0.46) global symptom burden. The burden of individual symptoms displayed similar patterns. Low (Cohen *d*, −0.60; 95% CI, −0.87 to −0.32) and moderate/high (*d*, −0.98; 95% CI, −1.53 to −0.43) general pain, moderate/high numbness (*d*, −0.99; 95% CI, −1.69 to −0.29), and moderate/high worry (*d*, −0.55; 95% CI, −0.99 to −0.11) were associated with lower HRQOL.

**Conclusions and Relevance:**

In this cross-sectional study of young childhood cancer survivors, symptom burden was prevalent. Caregiver anxiety and disparity-related neighborhood factors were associated with greater symptom burden, whereas meaning and purpose was a protective factor. Greater specific symptom burden contributed to poorer HRQOL. The findings suggest that interventions targeting resilience and neighborhood adversity may alleviate symptom burden and improve HRQOL.

## Key Points

**Question:**

What are the prevalence and factors associated with symptom burden in young (aged 8-18 years) childhood cancer survivors?

**Findings:**

In this cohort study of 302 young childhood cancer survivors, 38% of the survivors experienced moderate or high global cumulative symptom burden. Self-reported caregiver anxiety and neighborhood-level/census-track–based deprivation were associated with greater survivor-reported global symptom burden, while meaning and purpose was a protective factor.

**Meaning:**

The findings of this study suggest that symptoms are prevalent years after young childhood cancer survivors’ initial cancer diagnosis, and interventions to reduce caregiver anxiety and neighborhood adversity and improve resilience may alleviate symptom burden.

## Teaser

This cross-sectional study examines the factors associated with symptom burden and health-related quality of life in 5-year survivors of childhood cancer.

## Introduction

Pediatric cancer survivors often report various symptoms associated with treatment modalities or late effects, negatively impacting their health-related quality of life (HRQOL).^1,2^ However, existing patient-reported outcomes (PROs) research in pediatric cancer survivorship primarily targets adult survivors and neglects children and adolescents 5 years post diagnosis and younger than age 18 years.^3^

For pediatric cancer populations, symptoms can be assessed using the pediatric version of the Patient-Reported Outcomes Version of the Common Terminology Criteria for Adverse Events (Ped-PRO-CTCAE)^4,5,6,7^ to evaluate 3 attributes (frequency, severity, and interference with daily activities) for individual symptoms, resulting in 3 scores for each symptom.^8^ The Ped-PRO-CTCAE was initially developed to assess symptomatic adverse events (AEs) in patients with cancer undergoing therapies. This study applied the Ped-PRO-CTCAE to young cancer survivors to explore residual symptomatic AEs from cancer therapies and/or emerging treatment-induced symptomatic AEs after therapy completion (ie, late effects).

While a granular view of symptom attributes is valuable (eg, gaining insight into a patient’s pain severity), there are some instances where a global symptom burden metric is clinically useful (eg, treatment decision-making for a patient with high severity but low interference of pain). To our knowledge, characteristics of symptom burden, particularly contextual factors (eg, family dynamics, neighborhood environment), have not been examined in cancer survivors who are still children (aged 8-18 years). Previous studies using the Pediatric Quality of Life Cancer Module^9^ and the PediQUEST Memorial Symptom Assessment Scale^10^ have identified some protective factors (eg, resilience) associated with symptomatic AEs among children with cancer undergoing therapies, but efforts to identify protective factors for symptoms are presently lacking for young cancer survivors.

This study reports the prevalence of symptom severity and interference self-reported by young childhood cancer survivors using the Ped-PRO-CTCAE. Global cumulative and individual symptom burden metrics were developed by integrating symptom severity and interference attributes. Diverse characteristics, encompassing survivor’s personal and contextual factors, were considered in associations with symptom burden. Subsequently, the association between symptom burden and HRQOL was analyzed. We hypothesized that caregiver factors (eg, anxiety), neighborhood deprivation, and survivors’ meaning/purpose may be associated with cumulative burden across symptoms. Additionally, greater burden for some symptoms (eg, worry, fatigue) may be associated with poorer HRQOL.

## Methods

### Study Sample

Participants were 302 dyads of 5-year childhood cancer survivors who were previously treated at St Jude Children’s Research Hospital, enrolled in the St Jude Lifetime Cohort Study^11^ and aged 8 to 18 years at the time of assessment, and their primary caregivers (eFigure 1 in Supplement 1 provides the flow diagram). All eligible participants were approached and recruited during regular follow-ups in the hospital between November 1, 2017, and January 31, 2019. This study adhered to the Strengthening the Reporting of Observational Studies in Epidemiology (STROBE) reporting guideline. Self-reported paper-and-pencil surveys were administered to survivors and caregivers at St Jude’s survivorship clinic. Survivors self-reported their symptoms, HRQOL, and meaning/purpose; caregivers self-reported their anxiety, family conflict, and survivors’ and caregivers’ sociodemographic information. The study protocol was approved by St Jude’s Institutional Review Board; all participants provided written informed consent before data collection. Participants did not receive financial compensation for their participation in this ancillary study to the St Jude Lifetime Cohort Study.

### Measures

#### Symptoms

Using the 15-item core version of the Ped-PRO-CTCAE, 12 symptoms (stomach pain, constipation, mouth pain, nausea, fatigue, general pain, headache, numbness, worry, sadness, difficulty sleeping, and cough) containing the attributes of frequency, severity, and interference were used.^6,7^ Severity and interference attributes, more than frequency, were considered clinically meaningful and actionable. With comparable rates between severity and frequency attributes (eTable 1 in Supplement 1), we assessed the burden at the global and individual symptom levels using the combined severity and interference attributes of 12 symptoms. Each attribute was rated on a 4-point scale. To assess the burden of each symptom, responses were categorized as none if the survivor indicated that they did not have any severity or interference for that symptom (severity = 0 and interference = 0), low if their symptom was a little bad or the symptom had some interference (severity = 1 or interference = 1), or moderate/high if the symptom was bad or very bad or the symptom had a lot or a whole lot of interference (severity = 2 or 3 or interference = 2 or 3). To assess the global cumulative burden across 12 symptoms, the severity of each symptom was considered prevalent if the survivor reported at least a little severity (severity ≥1), and the number of symptoms with prevalent severity was counted to obtain a cumulative symptom severity score (0-12). The same dichotomization and count method was used for each symptom’s interference attribute. Following a review of item distributions and consensus among PRO methodologists (M.R.H., I.-C.H.), oncologists (G.T.A., M.M.H.), psychologists, a palliative care clinician (J.N.B.), and a statistician (D.K.S.), global cumulative burden was categorized as low if the survivor had 4 or fewer symptoms with prevalent severity and interference, moderate if 5 to 8 symptoms had either prevalent severity or interference, and high if 9 to 12 symptoms had either prevalent severity or interference, acknowledging the absence of a definitive high standard (eFigure 2 in Supplement 1 for scoring system).

#### Health-Related Quality of Life

Utility-based HRQOL was assessed with the EuroQol-5D Youth Report (EQ-5D-Y).^12^ The EQ-5D-Y is a measure of overall HRQOL that incorporates the general population’s preferences for health states into weights for individual EQ-5D-Y items. This measure encompasses 5 dimensions: mobility, taking care of oneself, doing usual activities, having pain or discomfort, and feeling worried, sad, or unhappy. The responses to 5 dimensions were weighted according to caregiver preferences gathered from a previous validation study and combined to calculate a HRQOL score (higher for better HRQOL).^13^

#### Cancer Diagnosis, Chronic Health Condition Burden, and Demographic Variables

Clinical information, including cancer diagnosis and time since diagnosis, was extracted from medical records. Survivorship clinicians assessed study participants for chronic health conditions (CHCs), graded as 1 (asymptomatic/mild), 2 (moderate), 3 (severe/disabling), or 4 (life-threatening) using a modified version of the Common Terminology Criteria for Adverse Events (CTCAE) grades.^14^ Individual CHCs were grouped by organ system, and each CHC group was assigned a composite grade based on the highest graded individual CHC within that organ system. Cardiovascular, endocrine, hematologic, and neurologic CHC groups were included in the multivariable analysis given their prevalence (n >30) and associations with global symptom burden in bivariate analysis (*P* < .20). Caregivers reported sociodemographic information, including survivors’ age at assessment (continuous in years), sex (male/female), race (American Indian, Asian, Black, White, more than 1, or unknown), ethnicity (Hispanic/non-Hispanic), primary caregivers’ age (continuous in years), mother’s educational level (below high school, high school graduate or General Educational Development, some college, college graduate, or post graduate), insurance status (private, public, or uninsured), annual household income (≤$19 999, $20 000-$54 999, or ≥$55 000), and residential address. Race and ethnicity were collected as part of the standard sociodemographic variables included in the St Jude Lifetime Cohort Study to examine the demographic variation in participants, but these variables were not included as covariates in the multivariable models.

#### Contextual Variables

Caregivers completed the 9-item family conflict domain of the Family Environment Scale.^15^ Raw sum scores were converted to T scores with a mean (SD) of 50 (10). Scores were converted to a *z* score (higher for greater conflict).

Census tract–based Social Vulnerability Index (SVI) measured neighborhood adversity per the survivor’s residential address.^16^ We used the overall SVI score, which encompasses neighborhood adversity across socioeconomic status, household composition, racial and ethnic minority status and language, and housing and transportation domains. Scores were dichotomized to compare survivors living in high deprivation areas (≥90th percentile of the SVI) with those living in low deprivation areas (<90th percentile of the SVI).

Caregiver’s anxiety was self-reported using the 6-item PROMIS Anxiety Short-Form^17^ to capture feelings of unease, fear, and worry. Raw sum scores were converted to T scores with a mean (SD) of 50 (10).^18^ Scores were converted to a *z* score (higher for greater anxiety).

#### Meaning/Purpose

Survivors completed the PROMIS Meaning and Purpose scale to assess feelings of hopefulness, optimism, and sense of life purpose.^19^ Raw sum scores were converted to T scores.^18^ Scores were converted to a *z* score (higher for more meaning/purpose).

### Statistical Analysis

Data analysis was conducted from March 13, 2023, to February 29, 2024. Descriptive statistics included frequency distributions of categorical variables and means (SDs) of continuous variables. The amount of missing data for all variables was below 10%, so available data analysis was used in all analyses. Two multinomial logistic regression models analyzed characteristics of global cumulative symptom burden and burden of each symptom. Model 1 included personal risk factors (eg, survivor’s age and diagnosis)^20,21^ and contextual risk factors (ie, family conflict, neighborhood deprivation, and caregiver anxiety) of greater symptom burden. Model 2 added survivor’s meaning/purpose to the risk factors in model 1. For each model, low symptom burden is the reference group. Multivariable linear regression with robust SEs, as recommended by EQ-5D-Y developers,^22,23^ assessed associations between symptom burden and HRQOL. LASSO methods were used to select individual symptoms for testing associations with HRQOL, while personal, contextual, and meaning/purpose factors were forced to stay in the model. Regression coefficients are presented as effect sizes (Cohen *d*), with 0.2 to 0.49 indicating small effects; moderate, 0.5 to 0.79; and large, greater than or equal to 0.8.^24^ A 2-sided *P* value <.05 indicated statistical significance. All analyses were performed in Stata, version 18.0 (StataCorp LLC).^25^

## Results

### Participant Characteristics

The mean (SD) age of the survivors was 14.2 (2.9) years at the time of assessment and 10.9 (2.9) years since diagnosis; 149 (49.3%) were female and 153 (50.7%) were male; 3 (1.0%) were American Indian, 1 (0.3%) was Asian, 56 (18.5%) were Black, and 232 (76.8%) were White; 143 (47.3%) were diagnosed with solid tumors, 109 (36.1%) with hematologic cancer, and 41 (13.6%) with central nervous system tumors. Table 1 displays the characteristics of 302 survivors and their caregivers.

**Table 1.** Characteristics of Participants

Characteristic	Survivors, No. (%) (N = 302)
Age at assessment, mean (SD), y	14.2 (2.9)
Sex	
Male	153 (50.7)
Female	149 (49.3)
Race	
American Indian	3 (1.0)
Asian	1 (0.3)
Black	56 (18.5)
White	232 (76.8)
>1	8 (2.7)
Unknown	2 (0.7)
Ethnicity	
Hispanic	28 (9.3)
Non-Hispanic	274 (90.7)
Mother’s educational background	
Below high school	15 (5.0)
High school graduate/GED	37 (12.3)
Some college/training after high school	88 (29.1)
College graduate/postgraduate level	147 (48.7)
Caregiver’s age, mean (SD), y	41.4 (9.1)
Survivor’s health insurance status	
Private	183 (60.6)
Public	94 (31.1)
Uninsured	11 (3.6)
Annual household income, $	
≤19 999	31 (10.3)
20 000-54 999	87 (28.8)
≥55 000	165 (54.6)
Primary cancer diagnosis	
Acute lymphoblastic leukemia	74 (24.5)
Other leukemia	22 (7.3)
Hodgkin lymphoma	4 (1.3)
Non-Hodgkin lymphoma	9 (3.0)
Central nervous system	41 (13.6)
Sarcomas	23 (7.6)
Wilms tumor	25 (8.3)
Neuroblastoma	25 (8.3)
Retinoblastoma	56 (18.5)
Other solid tumors	14 (4.6)
Time since cancer diagnosis, mean (SD), y	10.9 (2.9)
Treatment	
Anthracyclines	162 (53.6)
Classic alkylating agents	171 (56.6)
Corticosteroids	96 (31.8)
High-dose cytarabine	51 (16.9)
High-dose methotrexate	87 (28.8)
Epipodophyllotoxins	90 (29.8)
Vincristine	195 (64.6)
Any radiation	84 (27.8)
Major surgery	219 (72.5)

Abbreviation: GED, General Educational Development.

### Prevalence of Symptom Burden

The Figure depicts the prevalence of severity and interference separately by 12 symptoms, the burden for each symptom combining severity and interference attributes, and the global cumulative burden across 12 symptoms. Regarding severity, the most prevalent symptoms were feeling tired (45.2%), headaches (43.6%), and difficulty sleeping (42.2%). For interference, the most prevalent symptoms were feeling tired (32.0%), headaches (27.2%), and difficulty sleeping (27.3%) (eTable 1 in Supplement 1 reports on 3 attributes of 12 symptoms). The prevalence of low global cumulative symptom burden across 12 symptoms was 62.0% (n = 186); moderate, 25.7% (n = 77); and high, 12.3% (n = 37) (Figure).

**Figure.**
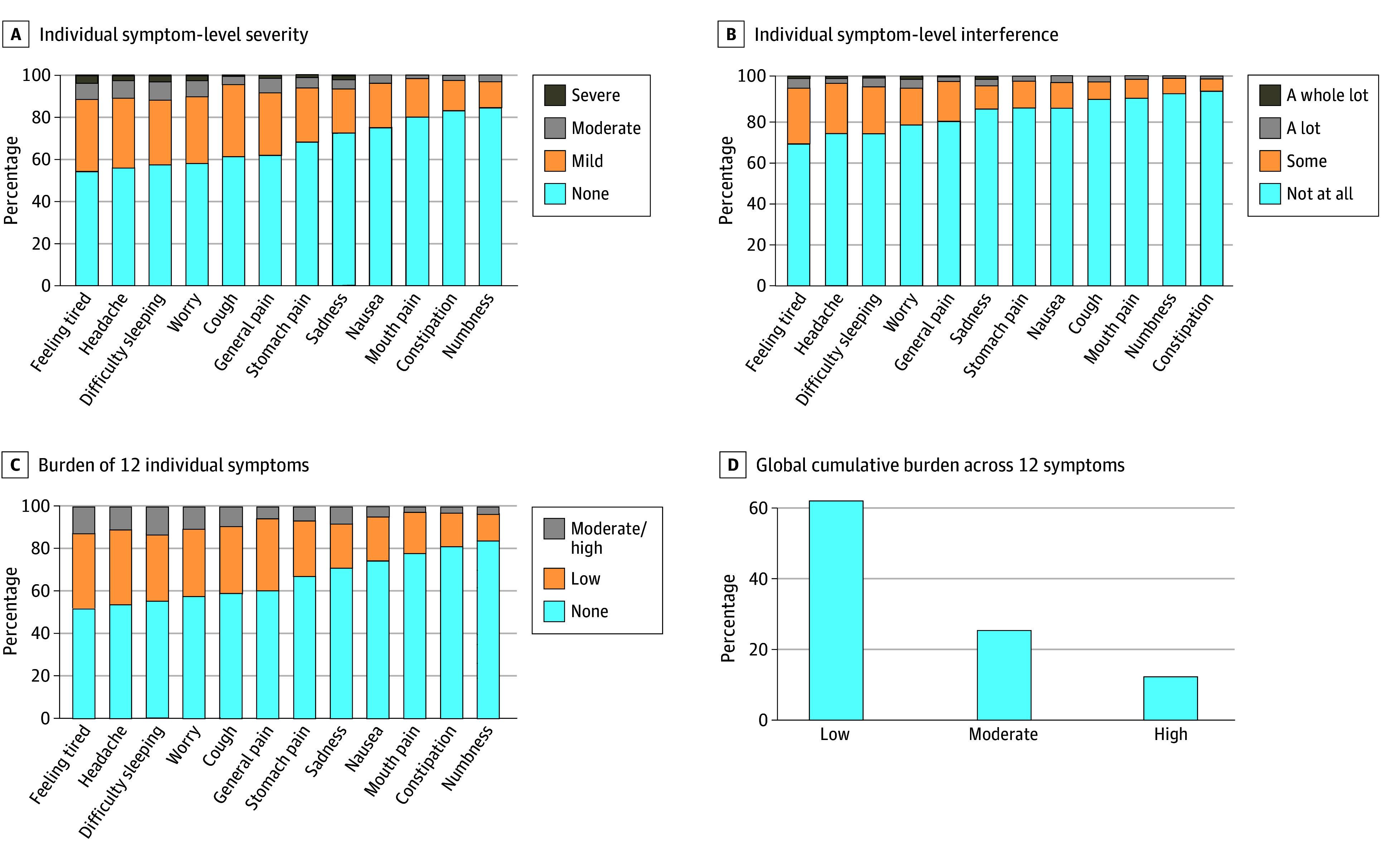
Prevalence of Symptom Severity, Symptom Interference, Burden of 12 Individual Symptoms (Combined Severity and Interference), and Global Cumulative Burden Across All 12 Symptoms The percentages for all 4 categories for both severity and interference are reported in eTable 1 in Supplement 1. Scores of the individual attributes (A, B) were combined to generate burden of individual symptoms (C) and global cumulative burden across 12 symptoms (D). eFigure 2 in Supplement 1 reports the method used to generate burden of individual symptoms and global cumulative burden across 12 symptoms.

### Risk Factors Associated With Global Cumulative Symptom Burden

Having neurologic CHCs was associated with moderate (RR, 3.45; 95% CI, 1.56-7.59) and high (RR, 6.70; 95% CI, 2.36-19.07) global cumulative symptom burden for models with and without meaning/purpose (Table 2; model 2 results are reported throughout this section unless otherwise indicated). Having hematologic CHCs was associated with high global symptom burden (RR, 6.17; 95% CI, 1.95-19.52). Greater caregiver anxiety was associated with moderate (RR, 1.56; 95% CI, 1.09-2.24) global symptom burden. Greater neighborhood deprivation was associated with moderate global symptom burden (RR, 4.86; 95% CI, 1.29-18.26). Survivors with greater meaning/purpose were less likely to have moderate (RR, 0.42; 95% CI, 0.29-0.61) and high (RR, 0.27; 95% CI, 0.16-0.46) global symptom burden.

**Table 2.** Characteristics of Global Cumulative Burden Across 12 Symptoms: Multinomial Logistic Regression Analysis^a^

Factor	Model 1^b^	*P* value	Model 2^c^	*P* value
	RR (95% CI)		RR (95% CI)	
**Moderate vs low global cumulative symptom burden**				
Diagnosis				
Solid tumors	1 [Reference]	NA	1 [Reference]	NA
CNS tumors	0.76 (0.28-2.10)	.60	0.66 (0.23-1.92)	.44
Hematologic cancer	0.74 (0.37-1.47)	.39	0.62 (0.30-1.29)	.20
Time since diagnosis	1.49 (0.71-3.14)	.29	2.01 (0.91-4.44)	.09
Sex				
Male	1 [Reference]	NA	1 [Reference]	NA
Female	1.31 (0.70-2.45)	.40	1.55 (0.80-3.03)	.20
Age	0.72 (0.34-1.50)	.37	0.53 (0.24-1.17)	.12
Mother’s educational level				
College graduate/postgraduate level	1 [Reference]	NA	1 [Reference]	NA
Below college graduate	0.81 (0.41-1.59)	.54	0.72 (0.35-1.48)	.37
Survivor insurance status				
Public or no health insurance	0.49 (0.23-1.03)	.06	0.40 (0.18-0.89)	.02
Private insurance	1 [Reference]	NA	1 [Reference]	NA
Chronic health conditions				
Cardiovascular	0.80 (0.42-1.52)	.49	0.77 (0.39-1.53)	.46
None	1 [Reference]	NA	1 [Reference]	NA
Endocrine	0.65 (0.34-1.27)	.21	0.61 (0.30-1.23)	.16
None	1 [Reference]	NA	1 [Reference]	NA
Hematologic	1.23 (0.46-3.27)	.67	1.46 (0.53-4.02)	.46
None	1 [Reference]	NA	1 [Reference]	NA
Neurologic	2.80 (1.34-5.86)	.01	3.45 (1.56-7.59)	.002
None	1 [Reference]	NA	1 [Reference]	NA
Family conflict	1.31 (0.95-1.80)	.10	1.23 (0.88-1.73)	.22
Area deprivation				
>90th percentile SVI	3.30 (0.95-11.48)	.06	4.86 (1.29-18.26)	.02
≤90th percentile SVI	1 [Reference]	NA	1 [Reference]	NA
Caregiver anxiety	1.64 (1.16-2.30)	.005	1.56 (1.09-2.24)	.01
Survivor’s meaning/purpose	NA	NA	0.42 (0.29-0.61)	<.001
**High vs low global cumulative symptom burden**				
Diagnosis				
Solid tumors	1 [Reference]	NA	1 [Reference]	NA
CNS tumors	0.89 (0.21-3.79)	.88	0.80 (0.17-3.75)	.78
Hematologic cancer	1.80 (0.69-4.71)	.23	1.39 (0.49-3.89)	.54
Time since diagnosis	1.05 (0.37-2.94)	.93	1.76 (0.56-5.48)	.33
Sex				
Female	1.43 (0.59-3.51)	.43	1.86 (0.69-4.97)	.22
Male	1 [Reference]	NA	1 [Reference]	NA
Age	0.90 (0.32-2.52)	.84	0.53 (0.17-1.66)	.28
Mother’s educational level				
College graduate/postgraduate level	1 [Reference]	NA	1 [Reference]	NA
Below college graduate	0.52 (0.19-1.41)	.20	0.44 (0.15-1.31)	.14
Survivor insurance status				
Public or no health insurance	0.77 (0.29-2.06)	.60	0.51 (0.17-1.51)	.22
Private insurance	1 [Reference]	NA	1 [Reference]	NA
Chronic health conditions				
Cardiovascular	1.12 (0.45-2.81)	.81	1.01 (0.37-2.72)	.99
None	1 [Reference]	NA	1 [Reference]	NA
Endocrine	1.24 (0.50-3.10)	.64	1.11 (0.41-2.99)	.84
None	1 [Reference]	NA	1 [Reference]	NA
Hematologic	5.09 (1.75-14.79)	.003	6.17 (1.95-19.52)	.002
None	1 [Reference]	NA	1 [Reference]	NA
Neurologic	4.71 (1.83-12.12)	.001	6.70 (2.36-19.07)	<.001
None	1 [Reference]	NA	1 [Reference]	NA
Family conflict	1.47 (0.95-2.29)	.08	1.46 (0.90-2.36)	.13
Area deprivation				
>90th percentile SVI	2.67 (0.54-13.32)	.23	5.09 (0.89-29.05)	.07
≤90th percentile SVI	1 [Reference]	NA	1 [Reference]	NA
Caregiver anxiety	1.30 (0.82-2.05)	.26	1.28 (0.79-2.09)	.31
Survivor’s meaning/purpose	NA	NA	0.27 (0.16-0.46)	<.001

Abbreviations: CNS, central nervous system; NA, not applicable; RR, risk ratio; SVI, Social Vulnerability Index.

^a^ Cancer treatment was not included because treatment is highly correlated with chronic health conditions.

^b^ Model 1 includes personal characteristics and contextual variables.

^c^ Model 2 includes personal characteristics, contextual variables, and personal meaning/purpose.

### Risk Factors Associated With Symptom Burden for Individual Symptoms

Table 3 presents the results of burden for 4 symptoms of interest (feeling tired, problems sleeping, worried/nervous, and general pain) selected to balance physical, psychological, and somatic symptoms. Having cardiovascular CHCs was associated with low burden of problems sleeping (RR, 2.07; 95% CI, 1.07-3.98). Having hematologic CHCs was also associated with moderate/high burden of feeling tired (RR, 5.34; 95% CI, 1.63-17.46) and worry (RR, 4.08; 95% CI, 1.07-15.52); having neurologic CHCs was associated with moderate/high symptom burden for all symptoms except stomach pain and constipation (eTable 2 in Supplement 1 for symptoms not reported in Table 3). Greater caregiver anxiety was associated with low burden for problems sleeping (RR, 1.58; 95% CI, 1.13-2.20). Greater neighborhood deprivation was associated with low worry (RR, 4.30; 95% CI, 1.32-14.04). More family conflict was associated with low problems sleeping (RR, 1.55; 95% CI, 1.13-2.13). Survivors with greater meaning/purpose were less likely to report low or moderate or high symptom burden for these 4 symptoms.

**Table 3.** Characteristics of Burden of 4 Selected Symptoms: Multinomial Logistic Regression Analysis^a^

Factor	RR (95% CI)															
	Feeling tired				Problems sleeping (trouble falling or staying asleep)				Worried or nervous				General pain			
	Model 1^b^	*P* value	Model 2^c^	*P* value	Model 1^b^	*P* value	Model 2^c^	*P* value	Model 1^b^	*P* value	Model 2^c^	*P* value	Model 1^b^	*P* value	Model 2^c^	*P* value
**Low vs no symptom burden**																
Diagnosis																
Solid tumors	1 [Reference]	NA	1 [Reference]	NA	1 [Reference]	NA	1 [Reference]	NA	1 [Reference]	NA	1 [Reference]	NA	1 [Reference]	NA	1 [Reference]	NA
CNS tumors	1.32 (0.54-3.24)	.54	1.28 (0.50-3.29)	.61	1.30 (0.50-3.43)	.59	1.25 (0.47-3.34)	.66	1.18 (0.45-3.09)	.74	1.15 (0.43-3.05)	.78	0.49 (0.19-1.29)	.15	0.44 (0.16-1.20)	.11
Hematologic cancer	1.15 (0.61-2.18)	.66	1.03 (0.53-2.02)	.92	1.64 (0.83-3.24)	.15	1.53 (0.77-3.05)	.23	1.49 (0.77-2.86)	.23	1.40 (0.72-2.71)	.32	0.55 (0.29-1.06)	.08	0.48 (0.24-0.95)	.03
Time since diagnosis	0.88 (0.45-1.74)	.72	1.06 (0.52-2.17)	.86	0.90 (0.44-1.83)	.77	1.04 (0.50-2.15)	.92	0.77 (0.39-1.55)	.47	0.85 (0.42-1.73)	.66	1.21 (0.61-2.40)	.59	1.46 (0.71-2.98)	.30
Sex																
Female	1.20 (0.67-2.13)	.54	1.39 (0.76-2.56)	.28	1.01 (0.54-1.87)	.98	1.08 (0.58-2.02)	.81	2.03 (1.12-3.66)	.02	2.19 (1.20-3.99)	.01	1.39 (0.77-2.52)	.27	1.64 (0.88-3.04)	.12
Male sex	1 [Reference]	NA	1 [Reference]	NA	1 [Reference]	NA	1 [Reference]	NA	1 [Reference]	NA	1 [Reference]	NA	1 [Reference]	NA	1 [Reference]	NA
Age	1.29 (0.66-2.52)	.46	1.08 (0.53-2.20)	.83	1.14 (0.56-2.30)	.73	0.99 (0.48-2.03)	.97	1.37 (0.69-2.72)	.38	1.24 (0.61-2.50)	.55	0.87 (0.44-1.71)	.68	0.72 (0.35-1.46)	.36
Mother’s educational level																
College graduate/postgraduate level	1 [Reference]	NA	1 [Reference]	NA	1 [Reference]	NA	1 [Reference]	NA	1 [Reference]	NA	1 [Reference]	NA	1 [Reference]	NA	1 [Reference]	NA
Below college graduate	0.74 (0.40-1.38)	.34	0.71 (0.37-1.37)	.31	0.51 (0.26-1.01)	.05	0.51 (0.26-1.00)	.05	0.61 (0.32-1.17)	.14	0.60 (0.31-1.14)	.12	0.75 (0.39-1.42)	.38	0.72 (0.37-1.40)	.33
Survivor insurance status																
Public or no health insurance	0.59 (0.30-1.15)	.12	0.49 (0.24-1.02)	.05	0.97 (0.48-1.96)	.94	0.91 (0.44-1.86)	.79	0.74 (0.37-1.48)	.39	0.72 (0.36-1.44)	.35	0.67 (0.34-1.32)	.24	0.59 (0.29-1.19)	.14
Private insurance	1 [Reference]	NA	1 [Reference]	NA	1 [Reference]	NA	1 [Reference]	NA	1 [Reference]	NA	1 [Reference]	NA	1 [Reference]	NA	1 [Reference]	NA
Chronic health condition																
Cardiovascular	1.46 (0.81-2.64)	.21	1.43 (0.76-2.68)	.26	2.09 (1.10-3.97)	.03	2.07 (1.07-3.98)	.03	0.86 (0.47-1.58)	.63	0.86 (0.46-1.59)	.62	1.46 (0.79-2.70)	.23	1.40 (0.74-2.65)	.30
None	1 [Reference]	NA	1 [Reference]	NA	1 [Reference]	NA	1 [Reference]	NA	1 [Reference]	NA	1 [Reference]	NA	1 [Reference]	NA	1 [Reference]	NA
Endocrine	1.22 (0.67-2.22)	.52	1.15 (0.61-2.16)	.68	1.03 (0.54-1.96)	.93	0.98 (0.51-1.88)	.94	0.95 (0.51-1.78)	.87	0.92 (0.49-1.75)	.80	1.30 (0.70-2.39)	.40	1.20 (0.64-2.27)	.57
None	1 [Reference]	NA	1 [Reference]	NA	1 [Reference]	NA	1 [Reference]	NA	1 [Reference]	NA	1 [Reference]	NA	1 [Reference]	NA	1 [Reference]	NA
Hematologic	0.93 (0.37-2.35)	.88	0.98 (0.37-2.58)	.97	1.74 (0.73-4.15)	.21	1.77 (0.73-4.30)	.21	1.34 (0.55-3.27)	.52	1.40 (0.57-3.43)	.47	2.49 (1.08-5.75)	.03	2.58 (1.08-6.15)	.03
None	1 [Reference]	NA	1 [Reference]	NA	1 [Reference]	NA	1 [Reference]	NA	1 [Reference]	NA	1 [Reference]	NA	1 [Reference]	NA	1 [Reference]	NA
Neurologic	1.43 (0.70-2.96)	.33	1.64 (0.77-3.51)	.20	1.27 (0.60-2.69)	.54	1.32 (0.61-2.82)	.48	2.00 (0.99-4.08)	.06	2.09 (1.02-4.32)	.05	2.47 (1.23-4.97)	.01	2.83 (1.37-5.87)	.01
None	1 [Reference]	NA	1 [Reference]	NA	1 [Reference]	NA	1 [Reference]	NA	1 [Reference]	NA	1 [Reference]	NA	1 [Reference]	NA	1 [Reference]	NA
Family conflict	1.36 (1.00-1.84)	.05	1.28 (0.94-1.76)	.12	1.61 (1.17-2.20)	.003^d^	1.55 (1.13-2.13)	.01	1.13 (0.83-1.54)	.44	1.09 (0.80-1.49)	.59	1.23 (0.91-1.66)	.19	1.16 (0.85-1.58)	.36
Area deprivation																
>90th percentile SVI	1.77 (0.51-6.20)	.37	2.24 (0.59-8.51)	.24	1.02 (0.29-3.55)	.98	1.14 (0.31-4.10)	.85	4.16 (1.30-13.37)	.02	4.30 (1.32-14.04)	.02	1.68 (0.54-5.23)	.37	1.99 (0.61-6.49)	.25
≤90th percentile SVI	1 [Reference]	NA	1 [Reference]	NA	1 [Reference]	NA	1 [Reference]	NA	1 [Reference]	NA	1 [Reference]	NA	1 [Reference]	NA	1 [Reference]	NA
Caregiver anxiety	1.48 (1.08-2.03)	.01	1.41 (1.01-1.96)	.04	1.64 (1.17-2.28)	.004 ^d^	1.58 (1.13-2.20)	.01	1.41 (1.03-1.95)	.03	1.37 (0.99-1.89)	.05	1.31 (0.95-1.79)	.10	1.26 (0.91-1.74)	.17
Survivor’s meaning/purpose	NA	NA	0.47 (0.34-0.66)	<.001^d^	NA	NA	0.66 (0.48-0.92)	.01	NA	NA	0.71 (0.52-0.99)	.04	NA	NA	0.53 (0.38-0.73)	<.001^d^
**Moderate/high vs no symptom burden**																
Diagnosis																
None	1 [Reference]	NA	1 [Reference]	NA	1 [Reference]	NA	1 [Reference]	NA	1 [Reference]	NA	1 [Reference]	NA	1 [Reference]	NA	1 [Reference]	NA
CNS tumors	0.81 (0.20-3.32)	.77	0.71 (0.16-3.19)	.66	0.41 (0.09-1.81)	.24	0.37 (0.08-1.69)	.20	2.79 (0.76-10.20)	.12	2.69 (0.62-11.54)	.18	0.76 (0.16-3.65)	.73	0.67 (0.13-3.39)	.63
Hematologic cancer	1.17 (0.43-3.21)	.76	0.82 (0.28-2.44)	.72	0.82 (0.33-2.06)	.68	0.67 (0.25-1.76)	.41	1.55 (0.56-4.29)	.40	1.13 (0.36-3.57)	.84	0.91 (0.31-2.69)	.87	0.57 (0.17-1.86)	.35
Time since diagnosis	0.77 (0.27-2.18)	.62	1.08 (0.35-3.31)	.90	2.55 (0.82-7.90)	.11	3.24 (1.00-10.51)	.05	1.80 (0.60-5.42)	.30	3.28 (0.92-11.73)	.07	1.42 (0.43-4.67)	.57	2.06 (0.58-7.41)	.27
Sex																
Female	1.32 (0.52-3.32)	.56	1.47 (0.55-3.92)	.44	1.16 (0.49-2.75)	.73	1.18 (0.49-2.87)	.71	1.49 (0.59-3.73)	.40	1.61 (0.56-4.60)	.38	2.47 (0.87-6.98)	.09	2.88 (0.96-8.61)	.06
Male	1 [Reference]	NA	1 [Reference]	NA	1 [Reference]	NA	1 [Reference]	NA	1 [Reference]	NA	1 [Reference]	NA	1 [Reference]	NA	1 [Reference]	NA
Age	1.47 (0.52-4.16)	.47	1.09 (0.35-3.32)	.89	0.44 (0.14-1.37)	.16	0.35 (0.11-1.14)	.08	0.69 (0.23-2.07)	.51	0.39 (0.11-1.38)	.14	0.64 (0.19-2.11)	.46	0.44 (0.12-1.57)	.21
Mother’s educational level																
Graduate/postgraduate level	1 [Reference]	NA	1 [Reference]	NA	1 [Reference]	NA	1 [Reference]	NA	1 [Reference]	NA	1 [Reference]	NA	1 [Reference]	NA	1 [Reference]	NA
Below college graduate	1.29 (0.48-3.44)	.62	1.33 (0.47-3.81)	.59	1.16 (0.46-2.95)	.76	1.11 (0.42-2.90)	.83	0.50 (0.18-1.40)	.19	0.35 (0.11-1.13)	.08	0.95 (0.33-2.73)	.92	0.83 (0.27-2.55)	.74
Survivor insurance status																
Public or no health insurance	0.60 (0.22-1.61)	.31	0.44 (0.15-1.26)	.12	0.86 (0.33-2.20)	.75	0.73 (0.27-1.96)	.53	1.37 (0.50-3.78)	.54	1.01 (0.33-3.13)	.98	0.63 (0.21-1.89)	.41	0.44 (0.13-1.42)	.17
Private insurance	1 [Reference]	NA	1 [Reference]	NA	1 [Reference]	NA	1 [Reference]	NA	1 [Reference]	NA	1 [Reference]	NA	1 [Reference]	NA	1 [Reference]	NA
Chronic health condition																
Cardiovascular CHC	1.45 (0.57-3.69)	.44	1.42 (0.53-3.81)	.49	1.43 (0.60-3.40)	.41	1.45 (0.59-3.55)	.42	0.99 (0.39-2.51)	.98	0.86 (0.31-2.41)	.78	0.89 (0.33-2.42)	.82	0.89 (0.31-2.53)	.82
None	1 [Reference]	NA	1 [Reference]	NA	1 [Reference]	NA	1 [Reference]	NA	1 [Reference]	NA	1 [Reference]	NA	1 [Reference]	NA	1 [Reference]	NA
Endocrine	0.53 (0.20-1.41)	.20	0.43 (0.15-1.21)	.11	0.53 (0.22-1.30)	.17	0.46 (0.18-1.18)	.11	0.55 (0.21-1.45)	.22	0.43 (0.14-1.26)	.12	0.49 (0.17-1.46)	.20	0.49 (0.16-1.51)	.21
None	1 [Reference]	NA	1 [Reference]	NA	1 [Reference]	NA	1 [Reference]	NA	1 [Reference]	NA	1 [Reference]	NA	1 [Reference]	NA	1 [Reference]	NA
Hematologic	3.98 (1.32-12.01)	.01	5.34 (1.63-17.46)	.01	1.15 (0.33-3.99)	.83	1.26 (0.35-4.51)	.73	3.03 (0.93-9.84)	.07	4.08 (1.07-15.52)	.04	1.59 (0.43-5.91)	.49	1.95 (0.50-7.67)	.34
None	1 [Reference]	NA	1 [Reference]	NA	1 [Reference]	NA	1 [Reference]	NA	1 [Reference]	NA	1 [Reference]	NA	1 [Reference]	NA	1 [Reference]	NA
Neurologic	8.37 (3.18-22.02)	<.001^d^	11.85 (4.09-34.33)	<.001^d^	6.23 (2.53-15.37)	<.001^d^	7.48 (2.88-19.43)	<.001^d^	4.15 (1.59-10.86)	.004^d^	8.36 (2.69-25.97)	<.001^d^	7.13 (2.47-20.65)	<.001^d^	9.00 (2.91-27.82)	<.001^d^
None	1 [Reference]	NA	1 [Reference]	NA	1 [Reference]	NA	1 [Reference]	NA	1 [Reference]	NA	1 [Reference]	NA	1 [Reference]	NA	1 [Reference]	NA
Family conflict	1.63 (1.03-2.59)	.04	1.56 (0.95-2.56)	.08	1.51 (0.98-2.32)	.06	1.40 (0.89-2.21)	.14	1.49 (0.95-2.34)	.08	1.45 (0.86-2.44)	.17	1.28 (0.80-2.07)	.31	1.25 (0.75-2.08)	.38
Area deprivation																
>90th percentile SVI	2.91 (0.60-14.25)	.19	4.58 (0.83-25.25)	.08	0.96 (0.17-5.29)	.96	1.17 (0.20-7.00)	.86	0.64 (0.06-7.00)	.71	0.79 (0.06-10.46)	.86	0.61 (0.06-5.85)	.67	0.90 (0.09-9.40)	.93
≤90th percentile SVI	1 [Reference]	NA	1 [Reference]	NA	1 [Reference]	NA	1 [Reference]	NA	1 [Reference]	NA	1 [Reference]	NA	1 [Reference]	NA	1 [Reference]	NA
Caregiver anxiety	1.40 (0.88-2.23)	.16	1.35 (0.82-2.23)	.24	1.31 (0.83-2.06)	.25	1.23 (0.76-1.98)	.40	1.52 (0.92-2.49)	.10	1.43 (0.82-2.51)	.21	1.66 (0.99-2.78)	.05	1.64 (0.95-2.84)	.08
Survivor’s meaning/purpose	NA	NA	0.31 (0.18-0.52)	<.001^d^	NA	NA	0.46 (0.29-0.72)	<.001^d^	NA	NA	0.22 (0.12-0.40)	<.001^d^	NA	NA	0.31 (0.17-0.56)	<.001^d^

Abbreviations: CHC, chronic health condition; CNS, central nervous system; NA, not applicable; RR, risk ratio; SVI, Social Vulnerability Index.

^a^ eTable 2 in Supplement 1 provides the remaining symptoms. Cancer treatment was not included because treatment is highly correlated with chronic health conditions.

^b^ Model 1 includes personal characteristics and contextual variables.

^c^ Model 2 includes personal characteristics, contextual variables, and personal meaning/purpose.

^d^ Significant when corrected for multiple comparisons, using Bonferroni correction (*P* = .004).

### Associations of Symptom Burden With HRQOL

At the global cumulative symptom burden level, survivors having moderate (*d*, −0.72; 95% CI, −1.00 to −0.44) or high (*d*, −1.37; 95% CI, −1.77 to −0.96) global burden were less likely to report higher HRQOL. To examine this association at the symptom level, 10 individual symptoms were selected into the model through LASSO methods (Table 4). Low (*d*, −0.60; 95% CI, −0.87 to −0.32) and moderate/high (*d*, −0.98; 95% CI, −1.53 to −0.43) general pain was associated with lower HRQOL by medium and large effects. Having moderate/high numbness (*d*, −0.99; 95% CI, −1.69 to −0.29) and moderate/high constipation (*d*, −0.71; 95% CI, −1.41 to −0.02) were associated with lower HRQOL by large and medium effects. Having moderate/high worry (*d*, −0.55; 95% CI, −0.99 to −0.11) and moderate/high sadness (*d*, −0.59; 95% CI, −1.11 to −0.06) were associated with lower HRQOL by medium effects.

**Table 4.** Associations of Burden of 12 Individual Symptoms and Utility-Based HRQOL: Multivariable Linear Regression Analysis^a^

Characteristic^b^	Utility-based HRQOL^c^		
	Unstandardized regression coefficient (95% CI)	Cohen *d* (95% CI)^d^	*P* value
Diagnosis			
Solid tumors	1 [Reference]	1 [Reference]	NA
CNS tumors	−0.02 (−0.05 to 0.01)	−0.19 (−0.50 to 0.13)	.24
Hematologic cancer	0.00 (−0.02 to 0.03)	0.04 (−0.18 to 0.26)	.71
Time since diagnosis	−0.00 (−0.03 to 0.02)	−0.04 (−0.28 to 0.21)	.77
Sex			
Female	−0.01 (−0.03 to 0.01)	−0.07 (−0.28 to 0.14)	.53
Male	1 [Reference]	1 [Reference]	NA
Age at assessment	0.01 (−0.02 to 0.03)	0.07 (−0.17 to 0.31)	.56
Mother’s educational level			
College graduate/postgraduate level	1 [Reference]	1 [Reference]	NA
Below college graduate	0.00 (−0.02 to 0.02)	0.02 (−0.20 to 0.24)	.86
Survivor insurance status			
Public or no health insurance	−0.00 (−0.03 to 0.02)	−0.05 (−0.28 to 0.18)	.69
Private insurance	1 [Reference]	1 [Reference]	NA
Chronic health condition			
Cardiovascular	0.00 (−0.02 to 0.03)	0.05 (−0.16 to 0.26)	.67
None	1 [Reference]	1 [Reference]	NA
Endocrine	0.01 (−0.02 to 0.03)	0.06 (−0.15 to 0.27)	.59
None	1 [Reference]	1 [Reference]	NA
Hematologic	0.01 (−0.02 to 0.04)	0.14 (−0.16 to 0.44)	.37
None	1 [Reference]	1 [Reference]	NA
Neurologic	0.00 (−0.03 to 0.03)	0.02 (−0.26 to 0.29)	.91
None	1 [Reference]	1 [Reference]	NA
Family conflict	−0.01 (−0.02 to 0.00)	−0.06 (−0.17 to 0.05)	.26
Area deprivation			
>90th percentile SVI	−0.01 (−0.05 to 0.03)	−0.09 (−0.53 to 0.34)	.68
≤90th percentile SVI	1 [Reference]	1 [Reference]	NA
Caregiver anxiety	−0.01 (−0.02 to 0.00)	−0.08 (−0.20 to 0.04)	.19
Survivor’s meaning/purpose	0.00 (−0.01 to 0.01)	0.01 (−0.10 to 0.13)	.80
Feeling tired			
No symptom burden	1 [Reference]	1 [Reference]	NA
Low symptom burden	−0.02 (−0.05 to 0.00)	−0.23 (−0.51 to 0.04)	.10
Moderate/high symptom burden	−0.02 (−0.07 to 0.03)	−0.21 (−0.70 to 0.28)	.40
Problems sleeping (trouble falling or staying asleep)			
No symptom burden	1 [Reference]	1 [Reference]	NA
Low symptom burden	−0.02 (−0.04 to 0.01)	−0.16 (−0.43 to 0.11)	.25
Moderate/high symptom burden	−0.00 (−0.04 to 0.04)	−0.01 (−0.43 to 0.41)	.96
Worried or nervous			
No symptom burden	1 [Reference]	1 [Reference]	NA
Low symptom burden	0.01 (−0.02 to 0.04)	0.13 (−0.15 to 0.40)	.36
Moderate/high symptom burden	−0.06 (−0.10 to −0.01)	−0.55 (−0.99 to −0.11)	.01
General pain			
No symptom burden	1 [Reference]	1 [Reference]	NA
Low symptom burden	−0.06 (−0.09 to −0.03)	−0.60 (−0.87 to −0.32)	<.001
Moderate/high symptom burden	−0.10 (−0.16 to −0.04)	−0.98 (−1.53 to −0.43)	.001
Cough			
No symptom burden	1 [Reference]	1 [Reference]	NA
Low symptom burden	−0.01 (−0.03 to 0.01)	−0.11 (−0.34 to 0.11)	.32
Moderate/high symptom burden	0.04 (−0.01 to 0.09)	0.43 (−0.07 to 0.93)	.09
Stomach pain			
No symptom burden	1 [Reference]	1 [Reference]	NA
Low symptom burden	0.02 (−0.01 to 0.05)	0.23 (−0.08 to 0.53)	.15
Moderate/high symptom burden	−0.04 (−0.10 to 0.02)	−0.38 (−0.98 to 0.23)	.22
Sad or unhappy feelings			
No symptom burden	1 [Reference]	1 [Reference]	NA
Low symptom burden	−0.01 (−0.04 to 0.02)	−0.13 (−0.44 to 0.19)	.43
Moderate/high symptom burden	−0.06 (−0.11 to −0.01)	−0.59 (−1.11 to −0.06)	.03
Nausea			
No symptom burden	1 [Reference]	1 [Reference]	NA
Low symptom burden	−0.01 (−0.04 to 0.03)	−0.06 (−0.38 to 0.27)	.74
Moderate/high symptom burden	−0.01 (−0.08 to 0.06)	−0.12 (−0.84 to 0.60)	.75
Problems with not being able to poop			
No symptom burden	1 [Reference]	1 [Reference]	NA
Low symptom burden	−0.01 (−0.05 to 0.02)	−0.12 (−0.45 to 0.21)	.48
Moderate/high symptom burden	−0.07 (−0.14 to −0.00)	−0.71 (−1.41 to −0.02)	.05
Numbness or tingly feeling in your hands or feet			
No symptom burden	1 [Reference]	1 [Reference]	NA
Low symptom burden	0.01 (−0.03 to 0.04)	0.06 (−0.29 to 0.41)	.72
Moderate/high symptom burden	−0.10 (−0.17 to −0.03)	−0.99 (−1.69 to −0.29)	.01

Abbreviations: CNS, central nervous system; HRQOL, health-related quality of life; NA, not applicable; SVI, Social Vulnerability Index.

^a^ LASSO methods used for variable selection.

^b^ Personal, contextual, and personal meaning/purpose variables were forced to stay in the model.

^c^ *R*^2^ = 0.55.

^d^ Approximate Cohen *d* (bStdY from listcoef command in Stata).

## Discussion

Approximately 40% of young childhood cancer survivors had moderate or high global symptom burdens that were evident years beyond their initial cancer diagnosis. Greater caregiver anxiety and neighborhood deprivation were linked to greater symptom burden, suggesting that caregiver’s mental health and the context where survivors live may be critical to consider in their symptom management. Survivors’ perception of life’s meaning/purpose was associated with higher symptom burden both globally and for most symptoms.

Compared with a previous report that evaluated young patients 4 to 14 days after beginning a round of treatment for childhood leukemia and lymphoma,^26^ young survivors in this study reported lower prevalence for some symptoms (eg, constipation, mouth pain, and nausea). However, other symptoms (eg, headaches, worry) had a similar prevalence among patients and survivors. This finding suggests that symptoms may persist or newly emerge after therapy completion. We also found the prevalence of some CHCs was associated with increased symptom burden across different domains, suggesting the importance of early identification and intervention on CHCs to address symptom burden and improve HRQOL.

The incorporation of both severity and interference data into a single metric represents a new approach to understanding survivors’ symptom burden. While severity and interference attributes provide unique insights into individual symptoms of survivors, assessing these attributes separately provides a large volume of data that could overwhelm clinical decision-making. Cumulative burden metrics have served as the primary end point in randomized clinical trials involving adult-onset cancers for comparative effectiveness and cost-effectiveness analyses.^27,28^ Our suggested approach, which involves integrating severity and interference attributes, is applicable in scenarios in which interpreting a single score is advantageous (eg, clinical decision-making, policy decisions). The gain in interpretability with this new approach likely comes with a loss of information and statistical power, so the method of scoring the Ped-PRO-CTCAE must be chosen with these considerations in mind.

In one study of a small sample of young childhood acute lymphoblastic leukemia survivors, risk factors associated with symptom prevalence included lower maternal educational level and family cohesion and higher parental emotional distress and parental protective behavior.^29^ Our study, containing broader pediatric cancer diagnoses, also found caregiver anxiety and family conflict to be associated with moderate or high symptom burden for some symptoms more than cancer diagnosis or personal demographic characteristics. Caregivers of children with cancer have been reported to be more overprotective^30^ and experience elevated distress and anxiety^31^ than the general population. Using family-centered psychosocial interventions (eg, the Surviving Cancer Competently Intervention Program^32^) may reduce survivors’ symptom burden by addressing caregivers’ anxiety, distress, and family strain and, conversely, treating survivors’ symptoms may alleviate caregivers’ anxiety.

This study revealed that neighborhood social vulnerability was associated with symptom burden. This finding is of great importance for survivors because a disadvantaged social environment, characterized by factors such as low socioeconomic status, unstable housing, and transportation barriers, can affect survivors’ physical and psychological stress and biomarkers of oxidative stress inflammation.^33,34^ Survivors’ stress physiologic status impacts their disease trajectory from diagnosis into survivorship and is linked to poorer outcomes, such as graft-vs-host disease, disability, and cancer-related mortality.^35^ Unlike patients undergoing therapy who are tethered to the hospital, long-term survivors are closely tied to their community environment, rendering neighborhood adversity a major risk factor of symptom burden.

Greater meaning/purpose was associated with lower symptom burden both at the global and individual symptom levels. Finding meaning/purpose can be an indicator of psychological resilience.^36,37^ Survivors with resilience skills may experience the same symptoms but report less burden compared with survivors without resilience skills. It is common practice in survivorship care to address the negative risk factors of experienced symptoms, but positive psychological factors may not be as frequently emphasized. Finding meaning/purpose is a skill that can be taught and incorporated into interventions to promote resilience. Given the associations of resilience with other outcomes, including mortality,^38^ future studies may offer useful interventions (eg, Promoting Resilience In Stress Management intervention for patients with cancer^39^) to promote a mindset of resiliency to lessen symptom burden.

The coefficients for caregiver anxiety and family conflict were reduced after meaning/purpose was adjusted, suggesting that meaning/purpose is protective in the association between caregiver anxiety and family strain and higher global symptom burden. However, the associations of neighborhood adversity with symptom burden were strengthened after adjusting for meaning/purpose. Meaning/purpose is a personal-level characteristic that may impact personal and family adversity, but community-level factors, such as community cohesion, may positively affect neighborhood adversity^40^ more than personal-level positive factors.

Symptom burden was associated with poorer HRQOL. The EQ-5D-Y assesses HRQOL, concentrating on symptoms and functionality,^12^ unlike measures of life satisfaction or overall well-being that gauge overall life experiences.^41^ Thus, it is more attuned to health issues, such as symptom burden, rather than neighborhood/family and meaning/purpose factors associated with subjective well-being. We suggest incorporating symptom screening into routine survivorship care, followed by interventions targeting specific symptoms (eg, behavioral and nonpharmacologic interventions for pain^42^) to improve the HRQOL of young cancer survivors.

### Limitations

This study has some limitations. First, with a cross-sectional design, it is not possible to detect causal outcomes among risk factors, symptom burden, and HRQOL. Future longitudinal studies are needed to elucidate the temporal nature of these associations. Second, the generalizability of this study to other young survivors is limited because survivors were recruited from a single institution that specifically cares for pediatric patients with cancer and survivors. Third, the current Ped-PRO-CTCAE, which includes assessments more salient to the acute phase of treatment (eg, constipation, nausea), may not fully characterize symptomatic issues of young survivors many years after completion of therapy. Designing a survivor-specific Ped-PRO-CTCAE that includes unique symptoms experienced by young, long-term survivors of childhood cancer is needed.

## Conclusions

In this cross-sectional study of childhood cancer survivors aged 8 to 18 years, survivors experienced symptom burden at the global and individual symptom levels. Survivors embracing a life filled with meaning/purpose have a lower prevalence of moderate or high symptom burden. Interventions targeting specific symptoms, family dynamics, and positive psychology to promote resilience, and offering resources to cope with challenging physical environments in their neighborhoods could alleviate symptom burden and enhance HRQOL for young, long-term cancer survivors.

## Notes

Supplement 1. **eFigure 1.** Flow Diagram Describing Patients Enrolled in Current Study **eTable 1.** Prevalence of 12 Individual Symptoms by 3 Attributes **eFigure 2.** Method of Generating Symptom Burden at Global and Individual Symptom Levels **eTable 2.** Characteristics of Burden for Remaining Symptoms Not Included in Table 3: Multinomial Logistic Regression Analysis

Supplement 2.
Data Sharing Statement

## References

[1] Linder LA, Hooke MC. Symptoms in children receiving treatment for cancer—part II: pain, sadness, and symptom clusters. J Pediatr Oncol Nurs. 2019;36(4):262-279. doi:10.1177/1043454219849578 31307323 PMC7197222

[2] Shin H, Dudley WN, Bhakta N, . Associations of symptom clusters and health outcomes in adult survivors of childhood cancer: a report from the St Jude Lifetime Cohort Study. J Clin Oncol. 2023;41(3):497-507. doi:10.1200/JCO.22.00361 36166720 PMC9870227

[3] Murugappan MN, King-Kallimanis BL, Reaman GH, . Patient-reported outcomes in pediatric cancer registration trials: a US Food and Drug Administration perspective. J Natl Cancer Inst. 2022;114(1):12-19. doi:10.1093/jnci/djab087 33930159 PMC8755487

[4] McFatrich M, Brondon J, Lucas NR, . Mapping child and adolescent self-reported symptom data to clinician-reported adverse event grading to improve pediatric oncology care and research. Cancer. 2020;126(1):140-147. doi:10.1002/cncr.32525 31553494 PMC6906242

[5] Reeve BB, McFatrich M, Mack JW, . Validity and reliability of the Pediatric Patient-Reported Outcomes version of the Common Terminology Criteria for Adverse Events. J Natl Cancer Inst. 2020;112(11):1143-1152. doi:10.1093/jnci/djaa016 31999349 PMC7669229

[6] Reeve BB, McFatrich M, Pinheiro LC, . Eliciting the child’s voice in adverse event reporting in oncology trials: cognitive interview findings from the Pediatric Patient-Reported Outcomes version of the Common Terminology Criteria for Adverse Events initiative. Pediatr Blood Cancer. 2017;64(3):1-7. doi:10.1002/pbc.26261 27650708 PMC5301979

[7] Reeve BB, Withycombe JS, Baker JN, . The first step to integrating the child’s voice in adverse event reporting in oncology trials: a content validation study among pediatric oncology clinicians. Pediatr Blood Cancer. 2013;60(7):1231-1236. doi:10.1002/pbc.24463 23335328

[8] Hinds PS, Pinheiro LC, McFatrich M, . Recommended scoring approach for the pediatric patient-reported outcomes version of the Common Terminology Criteria for Adverse Events. Pediatr Blood Cancer. 2022;69(6):e29452. doi:10.1002/pbc.29452 34866311 PMC9038621

[9] Rosenberg AR, Bradford MC, McCauley E, . Promoting resilience in adolescents and young adults with cancer: results from the PRISM randomized controlled trial. Cancer. 2018;124(19):3909-3917. doi:10.1002/cncr.31666 30230531

[10] Wolfe J, Orellana L, Ullrich C, . Symptoms and distress in children with advanced cancer: prospective patient-reported outcomes from the PediQUEST study. J Clin Oncol. 2015;33(17):1928-1935. doi:10.1200/JCO.2014.59.1222 25918277 PMC4451175

[11] Howell CR, Bjornard KL, Ness KK, . Cohort profile: the St Jude Lifetime Cohort Study (SJLIFE) for paediatric cancer survivors. Int J Epidemiol. 2021;50(1):39-49. doi:10.1093/ije/dyaa203 33374007 PMC8453382

[12] Wille N, Badia X, Bonsel G, . Development of the EQ-5D-Y: a child-friendly version of the EQ-5D. Qual Life Res. 2010;19(6):875-886. doi:10.1007/s11136-010-9648-y 20405245 PMC2892611

[13] Roudijk B, Sajjad A, Essers B, Lipman S, Stalmeier P, Finch AP. A value set for the EQ-5D-Y-3L in the Netherlands. Pharmacoeconomics. 2022;40(suppl 2):193-203. doi:10.1007/s40273-022-01192-0 36216977 PMC9549846

[14] Hudson MM, Ehrhardt MJ, Bhakta N, ; Jude Lifetime Cohort. Approach for classification and severity grading of long-term and late-onset health events among childhood cancer survivors in the St Jude Lifetime Cohort. Cancer Epidemiol Biomarkers Prev. 2017;26(5):666-674. doi:10.1158/1055-9965.EPI-16-0812 28035022 PMC5413397

[15] Moos RH, Moos BS. *Family Environment Scale Manual*. 4th ed. Mind Garden; 2009.

[16] Flanagan BE, Gregory EW, Hallisey EJ, Heitgerd JL, Lewis B. A Social Vulnerability Index for disaster management. J Homel Secur Emerg Manag. 2011;8(1):1-22. doi:10.2202/1547-7355.1792

[17] Pilkonis PA, Choi SW, Reise SP, Stover AM, Riley WT, Cella D; PROMIS Cooperative Group. Item banks for measuring emotional distress from the Patient-Reported Outcomes Measurement Information System (PROMIS): depression, anxiety, and anger. Assessment. 2011;18(3):263-283. doi:10.1177/1073191111411667 21697139 PMC3153635

[18] PROMIS Health Organization and PROMIS Cooperative Group. Calculate scores. Accessed August 24, 2023. https://www.healthmeasures.net/score-and-interpret/calculate-scores

[19] Forrest CB, Bevans KB, Filus A, . Assessing children’s eudaimonic well-being: the PROMIS pediatric meaning and purpose item banks. J Pediatr Psychol. 2019;44(9):1074-1082. doi:10.1093/jpepsy/jsz046 31233149 PMC6761958

[20] Horan MR, Srivastava DK, Bhakta N, . Determinants of health-related quality-of-life in adult survivors of childhood cancer: integrating personal and societal values through a health utility approach. EClinicalMedicine. 2023;58:101921. doi:10.1016/j.eclinm.2023.101921 37090443 PMC10114517

[21] Dixon SB, Liu Q, Chow EJ, . Specific causes of excess late mortality and association with modifiable risk factors among survivors of childhood cancer: a report from the Childhood Cancer Survivor Study cohort. Lancet. 2023;401(10386):1447-1457. doi:10.1016/S0140-6736(22)02471-0 37030315 PMC10149583

[22] Cham CH. Analysis of EQ-5D values. In: Devlin N, Parkin D, Janssen B, eds. Methods for Analysing and Reporting EQ-5D Data. Springer; 2020: chapter 4. https://www.ncbi.nlm.nih.gov/books/NBK565677/33347096

[23] Pullenayegum EM, Tarride JE, Xie F, Goeree R, Gerstein HC, O’Reilly D. Analysis of health utility data when some subjects attain the upper bound of 1: are Tobit and CLAD models appropriate? Value Health. 2010;13(4):487-494. doi:10.1111/j.1524-4733.2010.00695.x 20230549

[24] Cohen J. Statistical Power Analysis for the Behavioral Sciences. 2nd ed. Lawrence Erlbaum Associates; 1988.

[25] Stata Statistical Software. Release 18. StataCorp LP; 2023.

[26] Jacobs SS, Withycombe JS, Castellino SM, . Longitudinal use of patient reported outcomes in pediatric leukemia and lymphoma reveals clinically relevant symptomatic adverse events. Pediatr Blood Cancer. 2022;69(12):e29986. doi:10.1002/pbc.29986 36151978

[27] Maguire R, McCann L, Kotronoulas G, . Real time remote symptom monitoring during chemotherapy for cancer: European multicentre randomised controlled trial (eSMART). BMJ. 2021;374(1647):n1647. doi:10.1136/bmj.n1647 34289996 PMC8293749

[28] Karaaslan-Eşer A, Ayaz-Alkaya S. The effect of a mobile application on treatment adherence and symptom management in patients using oral anticancer agents: a randomized controlled trial. Eur J Oncol Nurs. 2021;52(101969):101969. doi:10.1016/j.ejon.2021.101969 33991868

[29] Huang IC, Brinkman TM, Mullins L, . Child symptoms, parent behaviors, and family strain in long-term survivors of childhood acute lymphoblastic leukemia. Psychooncology. 2018;27(8):2031-2038. doi:10.1002/pon.4769 29772082 PMC6298222

[30] Ernst M, Brähler E, Klein EM, . Parenting in the face of serious illness: childhood cancer survivors remember different rearing behavior than the general population. Psychooncology. 2019;28(8):1663-1670. doi:10.1002/pon.5138 31145818

[31] Sultan S, Leclair T, Rondeau É, Burns W, Abate C. A systematic review on factors and consequences of parental distress as related to childhood cancer. Eur J Cancer Care (Engl). 2016;25(4):616-637. doi:10.1111/ecc.12361 26354003 PMC5049674

[32] Kazak AE, Alderfer MA, Streisand R, . Treatment of posttraumatic stress symptoms in adolescent survivors of childhood cancer and their families: a randomized clinical trial. J Fam Psychol. 2004;18(3):493-504. doi:10.1037/0893-3200.18.3.493 15382974

[33] Leni Z, Künzi L, Geiser M. Air pollution causing oxidative stress. Curr Opin Toxicol. 2020;20-21:1-8. doi:10.1016/j.cotox.2020.02.006

[34] Bikomeye JC, Beyer AM, Kwarteng JL, Beyer KMM. Greenspace, inflammation, cardiovascular health, and cancer: a review and conceptual framework for greenspace in cardio-oncology research. Int J Environ Res Public Health. 2022;19(4):1-22. doi:10.3390/ijerph19042426 35206610 PMC8872601

[35] Taylor MR, Knight JM, Rosenberg AR. The biology of stress in cancer: applying the biobehavioral framework to adolescent and young adult oncology research. Brain Behav Immun Health. 2021;17:100321. doi:10.1016/j.bbih.2021.100321 34589815 PMC8474169

[36] Ostafin BD, Proulx T. Meaning in life and resilience to stressors. Anxiety Stress Coping. 2020;33(6):603-622. doi:10.1080/10615806.2020.1800655 32755239

[37] Southwick SM, Charney DS. Resilience: The Science of Mastering Life’s Greatest Challenges. Cambridge University Press; 2012. doi:10.1017/CBO9781139013857

[38] Nishimi K, Bürgin D, O’Donovan A. Psychological resilience to lifetime trauma and risk for cardiometabolic disease and mortality in older adults: a longitudinal cohort study. J Psychosom Res. 2023;175:111539. doi:10.1016/j.jpsychores.2023.111539 PMC12306198 39491282

[39] Rosenberg AR, Bradford MC, Barton KS, . Hope and benefit finding: results from the PRISM randomized controlled trial. Pediatr Blood Cancer. 2019;66(1):e27485. doi:10.1002/pbc.27485 30270489 PMC6249081

[40] Pei F, Li Z, Maguire-Jack K, Li X, Kleinberg J. Changes of perceived neighbourhood environment: a longitudinal study of collective efficacy among vulnerable families. Health Soc Care Community. 2022;30(6):e6228-e6239. doi:10.1111/hsc.14060 36178307

[41] National Research Council. Subjective Well-Being: Measuring Happiness, Suffering, and Other Dimensions of Experience. The National Academies Press; 2013:204. 24432436

[42] Schulte FSM, Patton M, Alberts NM, . Pain in long-term survivors of childhood cancer: a systematic review of the current state of knowledge and a call to action from the Children’s Oncology Group. Cancer. 2021;127(1):35-44. doi:10.1002/cncr.33289 33112416 PMC7875461

