# Supplementary material for: Multilevel Characteristics of Cumulative Symptom Burden in Young Survivors of Childhood Cancer
Source: JAMA Netw Open. 2024 May 7;7(5):e2410145. doi: 10.1001/jamanetworkopen.2024.10145 (PMC11077392; doi:10.1001/jamanetworkopen.2024.10145)
Supplement: Supplement 1. — eFigure 1. Flow Diagram Describing Patients Enrolled in Current Study eTable 1. Prevalence of 12 Individual Symptoms by 3 Attributes eFigure 2. Method of Generating Symptom Burden at Global and Individual Symptom Levels eTable 2. Characteristics of Burden for Remaining Symptoms Not Included in Table 3: Multinomial Logistic Regression Analysis [file jamanetwopen-e2410145-s001.pdf]

Supplementary Online Content

Horan MR, Srivastava DK, Choi J, et al. Multilevel characteristics of cumulative symptom burden in young survivors of childhood cancer. *JAMA Netw Open*. 2024;7(5):e2410145. doi:10.1001/jamanetworkopen.2024.10145

eFigure 1. Flow Diagram Describing Patients Enrolled in Current Study.

eTable 1. Prevalence of 12 Individual Symptoms by 3 Attributes

eFigure 2. Method of Generating Symptom Burden at Global and Individual Symptom Levels

eTable 2. Characteristics of Burden for Remaining Symptoms Not Included in Table 3: Multinomial Logistic Regression Analysis

This supplementary material has been provided by the authors to give readers additional information about their work.

© 2024 Horan MR et al. *JAMA Network Open.*

eFigure 1. Flow diagram describing patients enrolled in current study.

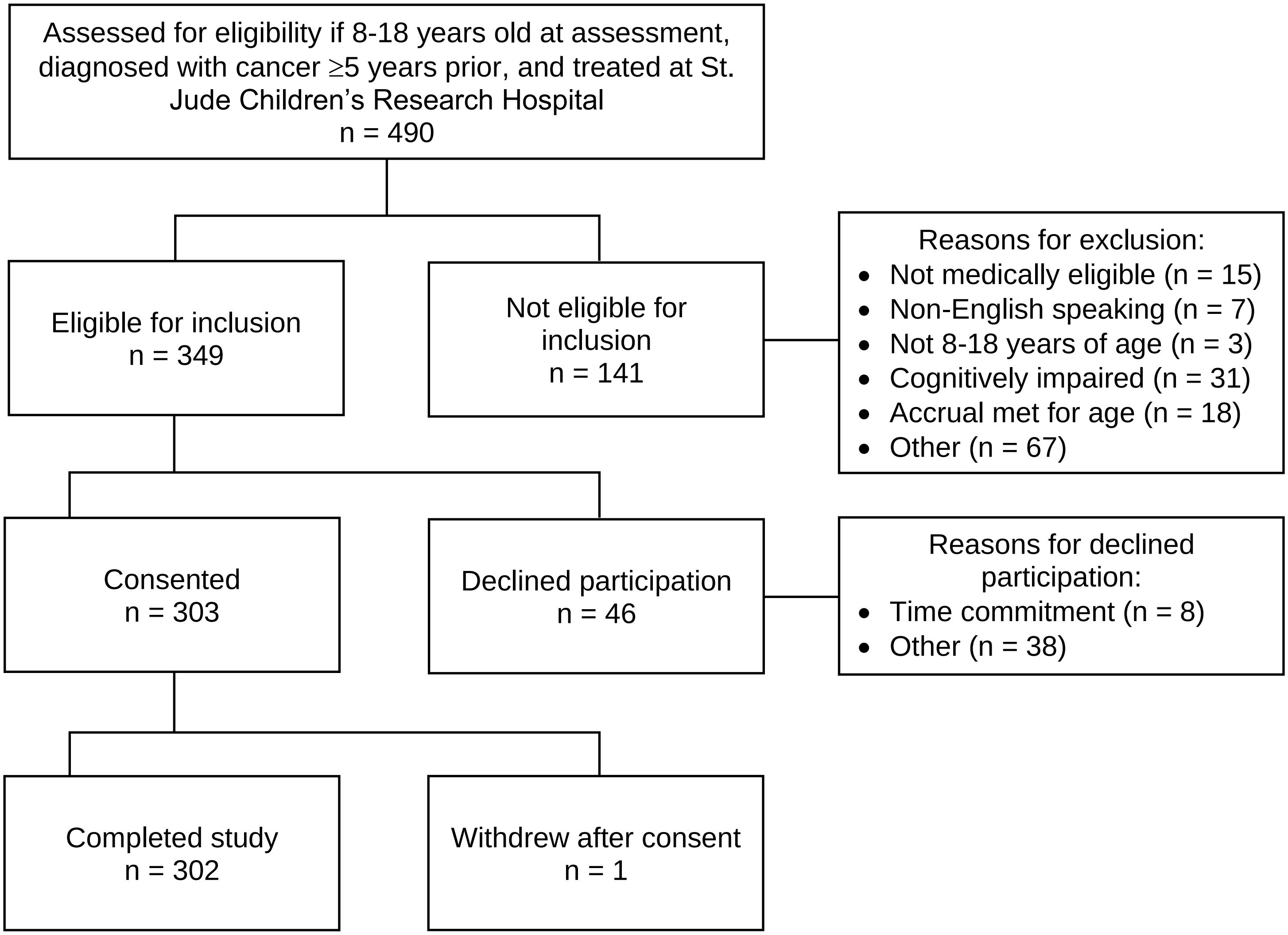

© 2024 Horan MR et al. *JAMA Network Open.*

eTable 1. Prevalence of 12 individual symptoms by 3 attributes

Attribute	Response	Feeling tired	Headache	Difficulty sleeping	Worry	Cough	General pain	Stomach pain	Sadness	Nausea	Mouth pain	Constipation	Numbness
Frequency [a, b]	Never	---	160, 53.2%	169, 56.2%	164, 54.5%	155, 52.0%	174, 57.8%	191, 63.5%	---	228, 75.8%	238, 79.1%	247, 82.1%	---
	Sometimes		119, 39.5%	97, 32.2%	104, 34.6%	124, 41.6%	109, 36.2%	100, 33.2%		64, 21.3%	56, 18.6%	48, 16.0%	
	Most of the time		17, 5.7%	22, 7.3%	21, 7.0%	14, 4.7%	16, 5.3%	8, 2.7%		8, 2.7%	4, 1.3%	6, 2.0%	
	Almost all the time		5, 1.7%	13, 4.3%	12, 4.0%	5, 1.7%	2, 0.7%	2, 0.7%		1, 0.3%	3, 1.0%	0, 0.0%	
Severity	None	165, 54.8%	169, 56.3%	174, 57.8%	176, 58.5%	186, 61.8%	188, 62.5%	207, 68.8%	219, 72.8%	227, 75.4%	241, 80.1%	251, 83.4%	255, 84.7%
	Mild	102, 33.9%	99, 33.0%	92, 30.6%	95, 31.6%	102, 33.9%	88, 29.2%	76, 25.3%	63, 20.9%	63, 20.9%	55, 18.3%	43, 14.3%	38, 12.6%
	Moderate	23, 7.6%	25, 8.3%	26, 8.6%	23, 7.6%	11, 3.7%	21, 7.0%	15, 5.0%	13, 4.3%	10, 3.3%	5, 1.7%	7, 2.3%	6, 2.0%
	Severe	11, 3.7%	7, 2.3%	9, 3.0%	7, 2.3%	2, 0.7%	4, 1.3%	3, 1.0%	6, 2.0%	1, 0.3%	0, 0.0%	0, 0.0%	2, 0.7%
Interference	Not at all	205, 68.1%	219, 72.8%	219, 72.8%	232, 77.1%	268, 89.0%	237, 78.7%	256, 85.1%	255, 84.7%	256, 85.1%	270, 89.7%	280, 93.0%	276, 91.7%
	Some	79, 26.3%	72, 23.9%	67, 22.3%	52, 17.3%	25, 8.3%	57, 18.9%	38, 12.6%	32, 10.6%	36, 12.0%	27, 9.0%	17, 5.7%	22, 7.3%
	A lot	14, 4.7%	7, 2.3%	12, 4.0%	13, 4.3%	8, 2.7%	6, 2.0%	7, 2.3%	10, 3.3%	8, 2.7%	2, 0.7%	4, 1.3%	2, 0.7%
	A whole lot	3, 1.0%	3, 1.0%	3, 1.0%	4, 1.3%	0, 0.0%	1, 0.3%	0, 0.0%	4, 1.3%	1, 0.3%	2, 0.7%	0, 0.0%	1, 0.3%

[a] Feeling tired, sadness, and numbness were not assessed for the frequency attribute
[b] Pearson correlations between frequency and severity for each symptom: stomach pain = 0.84, constipation = 0.79, mouth pain = 0.81, nausea = 0.86, general pain = 0.75, headache = 0.82, worry = 0.87, difficulty sleeping = 0.86, cough = 0.76

© 2024 Horan MR et al. *JAMA Network Open.*

eFigure 2. Method of generating symptom burden at global and individual symptom levels

A. Method of generating global cumulative burden across 12 symptoms

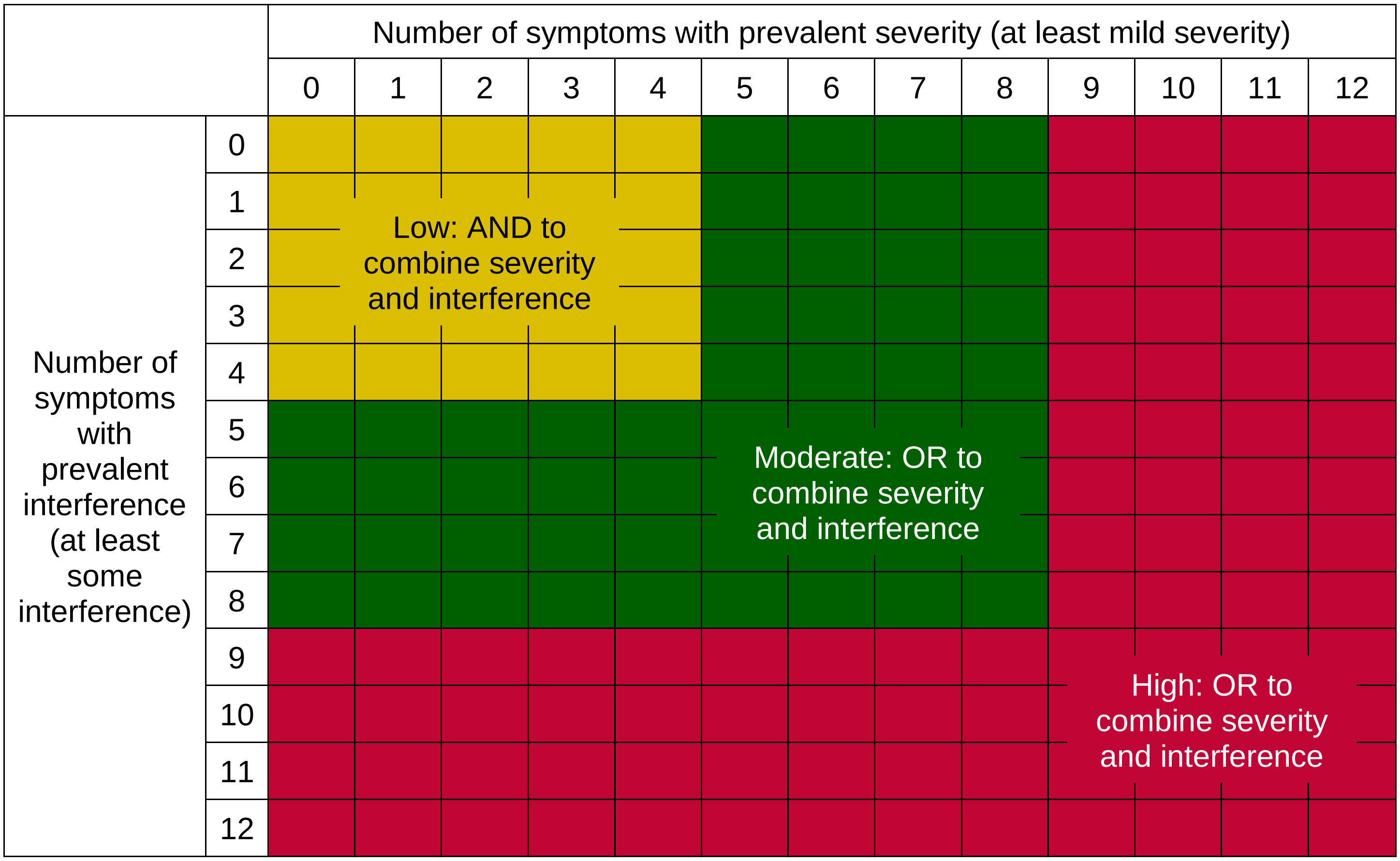

Yellow = low global cumulative burden (0-4 symptoms with endorsed severity and interference, 62% of survivors); green = moderate global cumulative burden (5-8 symptoms with endorsed severity or interference, 26%); red = high global cumulative burden (9-12 symptoms with endorsed severity or interference, 12%).

B. Method of generating burden of 12 individual symptoms

		Symptom severity			
		None	Mild	Moderate	Severe
Symptom interference	Not at all	None: AND to combine			
	Some		Low: OR to combine		
	A lot			Moderate/high: OR to combine	
	A whole lot				

Blue = no symptom burden (severity = “none” and interference = “not at all”); orange = low symptom burden (severity = “mild” and/or interference = “some”); gray = moderate/high symptom burden (severity = “moderate” or “severe” and/or interference = “a lot” or “a whole lot”)

© 2024 Horan MR et al. *JAMA Network Open.*

eTable 2. Characteristics of burden for remaining symptoms not included in Table 3: multinomial logistic regression analysis [a]

Determinants	Head hurt (headache)		Cough		Stomach pain		Sad or unhappy feelings	
	Model 1 [b]	Model 2 [c]	Model 1	Model 2	Model 1	Model 2	Model 1	Model 2
	RR [d] (95% CI), p	RR (95% CI), p	RR (95% CI), p	RR (95% CI), p	RR (95% CI), p	RR (95% CI), p	RR (95% CI), p	RR (95% CI), p
**Low symptom burden (Ref: No symptom burden)**								
Diagnosis (Ref: solid tumors)								
CNS [e] tumors	0.35 (0.13-0.96), .04	0.34 (0.12-0.93), .04	0.90 (0.38-2.11), .81	0.87 (0.37-2.07), .76	1.16 (0.43-3.10), .77	1.14 (0.42-3.09), .80	1.58 (0.57-4.38), .38	1.45 (0.48-4.36), .51
Hematologic cancer	0.71 (0.37-1.35), .29	0.69 (0.36-1.31), .26	0.86 (0.46-1.58), .62	0.82 (0.44-1.53), .54	1.47 (0.74-2.90), .27	1.38 (0.70-2.75), .36	1.73 (0.83-3.59), .14	1.37 (0.63-3.01), .43
Time since diagnosis	1.31 (0.66-2.59), .45	1.39 (0.69-2.80), .35	0.93 (0.49-1.75), .82	1.00 (0.53-1.91), 1.00	1.27 (0.61-2.65), .53	1.45 (0.68-3.09), .34	0.74 (0.35-1.58), .44	0.95 (0.42-2.16), .91
Female sex (Ref: Male)	1.32 (0.74-2.35), .35	1.38 (0.77-2.48), .28	0.67 (0.38-1.16), .15	0.70 (0.40-1.22), .20	2.27 (1.21-4.26), .01	2.53 (1.32-4.84), .01	1.47 (0.75-2.86), .26	1.77 (0.86-3.65), .12
Age	0.78 (0.40-1.54), .48	0.73 (0.37-1.46), .38	1.02 (0.55-1.92), .95	0.95 (0.50-1.80), .87	0.82 (0.39-1.71), .59	0.71 (0.33-1.52), .38	1.35 (0.63-2.87), .44	1.04 (0.46-2.35), .92
Mother's education (Ref: College graduate/post graduate level)	0.56 (0.30-1.07), .08	0.56 (0.30-1.07), .08	0.72 (0.40-1.31), .28	0.72 (0.40-1.29), .27	0.74 (0.38-1.44), .38	0.71 (0.36-1.41), .33	1.07 (0.53-2.16), .86	1.01 (0.47-2.16), .98
Survivor has public or no health insurance (Ref: Private insurance)	0.64 (0.32-1.26), .19	0.61 (0.30-1.21), .15	0.97 (0.52-1.80), .92	0.94 (0.50-1.76), .85	0.44 (0.21-0.95), .04	0.40 (0.19-0.88), .02	0.84 (0.40-1.77), .64	0.63 (0.28-1.43), .27
Cardiovascular CHC [f] (Ref: None)	0.79 (0.43-1.45), .44	0.76 (0.41-1.41), .39	0.86 (0.49-1.52), .60	0.83 (0.47-1.48), .53	0.91 (0.48-1.73), .78	0.87 (0.45-1.68), .69	0.88 (0.45-1.73), .71	0.82 (0.39-1.70), .58
Endocrine CHC (Ref: None)	2.74 (1.47-5.10), .002*	2.71 (1.45-5.06), .002*	1.23 (0.69-2.19), .48	1.20 (0.67-2.15), .53	1.34 (0.71-2.56), .37	1.34 (0.69-2.58), .39	0.70 (0.34-1.41), .31	0.63 (0.29-1.36), .24
Hematologic CHC (Ref: None)	1.50 (0.62-3.62), .36	1.48 (0.61-3.61), .38	1.56 (0.71-3.42), .27	1.52 (0.69-3.35), .30	1.85 (0.78-4.39), .17	1.82 (0.76-4.39), .18	1.06 (0.41-2.76), .91	1.13 (0.41-3.13), .82
Neurologic CHC (Ref: None)	2.95 (1.42-6.13), .004*	3.08 (1.47-6.47), .003*	1.16 (0.59-2.28), .68	1.20 (0.60-2.38), .60	0.89 (0.42-1.89), .76	0.92 (0.43-1.96), .82	1.67 (0.78-3.56), .19	2.03 (0.89-4.64), .09
Family conflict	0.97 (0.72-1.32), .87	0.94 (0.69-1.28), .71	1.13 (0.85-1.50), .40	1.10 (0.82-1.46), .53	1.25 (0.91-1.71), .17	1.21 (0.88-1.66), .25	1.17 (0.84-1.63), .35	1.12 (0.78-1.61), .53
High area deprivation (Ref: ≤90th percentile SVI [g])	0.93 (0.27-3.15), .90	0.96 (0.28-3.26), .95	1.71 (0.60-4.82), .31	1.79 (0.63-5.13), .28	5.47 (1.70-17.61), .004*	6.14 (1.87-20.12), .003*	0.37 (0.07-1.88), .23	0.42 (0.08-2.32), .32
Caregiver anxiety	1.32 (0.96-1.82), .09	1.30 (0.94-1.78), .11	1.16 (0.86-1.55), .33	1.13 (0.84-1.51), .43	1.40 (1.00-1.96), .05	1.36 (0.96-1.92), .08	1.72 (1.20-2.45), .003*	1.64 (1.12-2.40), .01
Survivor's meaning/purpose	---	0.79 (0.58-1.07), .13	---	0.77 (0.58-1.02), .07	---	0.66 (0.47-0.91), .01	---	0.35 (0.23-0.53), <.001*
**Moderate/high symptom burden (Ref: No symptom burden)**								
Diagnosis (Ref: solid tumors)								
CNS tumors	1.45 (0.35-5.97), .61	1.34 (0.32-5.64), .69	0.25 (0.02-2.87), .27	0.26 (0.02-2.95), .28	N/A	N/A	0.77 (0.17-3.61), .74	0.65 (0.13-3.37), .61
Hematologic cancer	1.12 (0.39-3.25), .83	0.78 (0.25-2.40), .67	1.31 (0.34-5.04), .70	1.19 (0.30-4.75), .80	1.12 (0.38-3.31), .84	0.98 (0.33-2.97), .98	0.88 (0.30-2.64), .82	0.70 (0.22-2.29), .56
Time since diagnosis	5.73 (1.40-23.36), .01	9.13 (1.95-42.72), .005	6.16 (0.63-60.51), .12	6.99 (0.70-69.72), .10	1.86 (0.45-7.67), .39	2.18 (0.52-9.21), .29	1.40 (0.43-4.53), .57	1.83 (0.52-6.49), .35
Female sex (Ref: Male)	1.57 (0.58-4.27), .38	1.61 (0.58-4.46), .36	1.52 (0.42-5.44), .52	1.42 (0.39-5.21), .60	3.90 (1.26-12.13), .02	4.09 (1.29-12.95), .02	0.99 (0.36-2.71), .98	1.00 (0.34-2.97), 1.00
Age	0.20 (0.05-0.79), .02	0.13 (0.03-0.58), .01	0.15 (0.02-1.49), .11	0.14 (0.01-1.35), .09	0.47 (0.11-1.93), .29	0.40 (0.09-1.70), .21	0.84 (0.26-2.71), .77	0.66 (0.19-2.32), 0.51

© 2024 Horan MR et al. *JAMA Network Open.*

Determinants	Head hurt (headache)		Cough		Stomach pain		Sad or unhappy feelings	
	Model 1 [b]	Model 2 [c]	Model 1	Model 2	Model 1	Model 2	Model 1	Model 2
	RR [d] (95% CI), p	RR (95% CI), p	RR (95% CI), p	RR (95% CI), p	RR (95% CI), p	RR (95% CI), p	RR (95% CI), p	RR (95% CI), p
**Moderate/high symptom burden (Ref: No symptom burden)**								
Mother's education (Ref: College graduate/post graduate level)	2.58 (0.84-7.94), .10	2.53 (0.81-7.95), .11	0.31 (0.06-1.52), .15	0.29 (0.06-1.48), .14	0.66 (0.20-2.13), .49	0.62 (0.19-2.05), .44	1.25 (0.42-3.67), .69	1.24 (0.39-3.91), .72
Survivor has public or no health insurance (Ref: Private insurance)	0.54 (0.19-1.55), .25	0.42 (0.14-1.25), .12	3.04 (0.69-13.40), .14	2.64 (0.57-12.25), .22	0.74 (0.22-2.41), .61	0.64 (0.19-2.17), .47	0.90 (0.30-2.71), .85	0.68 (0.21-2.19), .52
Cardiovascular CHC (Ref: None)	0.76 (0.29-2.04), .59	0.72 (0.26-1.98), .52	1.20 (0.32-4.50), .79	1.22 (0.32-4.64), .77	0.61 (0.21-1.74), .35	0.59 (0.20-1.73), .34	1.64 (0.58-4.64), .35	1.51 (0.50-4.56), .46
Endocrine CHC (Ref: None)	0.47 (0.16-1.38), .17	0.44 (0.15-1.34), .15	0.94 (0.26-3.45), .93	0.88 (0.24-3.23), .84	0.77 (0.25-2.37), .65	0.79 (0.25-2.46), .68	0.42 (0.14-1.21), .11	0.29 (0.09-0.93), .04
Hematologic CHC (Ref: None)	2.16 (0.62-7.50), .23	2.77 (0.75-10.25), .13	0.26 (0.03-2.55), .25	0.30 (0.03-2.85), .29	1.34 (0.31-5.73), .69	1.44 (0.34-6.18), .62	2.71 (0.80-9.22), .11	2.95 (0.81-10.73), .10
Neurologic CHC (Ref: None)	11.18 (3.98-31.48), <.001*	15.20 (5.00-46.26), <.001*	24.66 (5.60-108.57), <.001*	24.38 (5.59-106.26), <.001*	3.07 (0.98-9.61), .05	2.97 (0.95-9.35), .06	3.72 (1.34-10.34), .01	5.87 (1.90-18.19), .002*
Family conflict	1.11 (0.70-1.78), .66	1.02 (0.63-1.68), .92	1.30 (0.71-2.38), .40	1.32 (0.72-2.45), .37	1.14 (0.66-1.98), .64	1.12 (0.64-1.96), .69	1.30 (0.81-2.11), .28	1.17 (0.69-1.98), .56
High area deprivation (Ref: ≤90th percentile SVI)	1.33 (0.25-7.04), .73	2.03 (0.38-10.82), .41	N/A	N/A	1.50 (0.15-15.22), .73	1.76 (0.17-18.04), .64	0.48 (0.05-4.68), .53	0.54 (0.05-5.59), .61
Caregiver anxiety	2.12 (1.27-3.52), .004*	2.09 (1.23-3.55), .01	0.99 (0.51-1.92), .98	0.94 (0.47-1.85), .85	1.12 (0.64-1.95), .69	1.08 (0.62-1.91), .78	1.30 (0.76-2.23), .33	1.28 (0.72-2.28), .41
Survivor's meaning/purpose	---	0.45 (0.27-0.76), .003 *	---	0.64 (0.32-1.27), .20	---	0.59 (0.34-1.04), .07	---	0.29 (0.16-0.53), <.001*

[a] Cancer treatment was not included because treatment is highly correlated with CHCs. Models with mouth pain as the outcome had convergence issues and are not reported here.
[b] Model 1 = personal characteristics and contextual variables
[c] Model 2 = personal characteristics, contextual variables, and personal meaning/purpose
[d] RR = risk ratio
[e] CNS = central nervous system
[f] CHC = chronic health condition
[g] SVI = Social Vulnerability Index
N/A = empty cell
* Significant when corrected for multiple comparisons using Bonferroni's correction (p = 0.004)

© 2024 Horan MR et al. *JAMA Network Open.*

eTable 2 (continued). Determinants of burden for remaining symptoms not included in Table 3: multinomial logistic regression analysis a

Determinants	Feel sick to your stomach (nausea)		Problems with not being able to poop		Numbness or tingly feeling in your hands or feet	
	Model 1 [b]	Model 2 [c]	Model 1	Model 2	Model 1	Model 2
	RR [d] (95% CI), p	RR (95% CI), p	RR (95% CI), p	RR (95% CI), p	RR (95% CI), p	RR (95% CI), p
**Low symptom burden (Ref: No symptom burden)**						
Diagnosis (Ref: solid tumors)						
CNS [e] tumors	0.96 (0.33-2.80), .94	0.87 (0.29-2.62), .81	2.26 (0.69-7.44), .18	2.20 (0.67-7.23), .20	0.75 (0.21-2.61), .65	0.74 (0.21-2.58), .63
Hematologic cancer	1.74 (0.83-3.67), .14	1.52 (0.70-3.28), .29	2.76 (1.16-6.55), .02	2.62 (1.10-6.24), .03	0.70 (0.26-1.88), .48	0.66 (0.24-1.79), .41
Time since diagnosis	0.79 (0.36-1.72), .55	0.96 (0.43-2.17), .92	0.90 (0.36-2.27), .83	0.95 (0.37-2.41), .91	0.98 (0.37-2.63), .97	1.11 (0.40-3.05), .84
Female sex (Ref: Male)	1.11 (0.56-2.18), .77	1.22 (0.61-2.48), .57	1.91 (0.87-4.18), .11	1.95 (0.88-4.29), .10	0.82 (0.35-1.95), .66	0.85 (0.36-2.01), .70
Age	1.27 (0.58-2.75), .55	1.04 (0.46-2.34), .93	0.99 (0.40-2.49), .99	0.94 (0.37-2.38), .90	0.94 (0.35-2.52), .90	0.84 (0.30-2.29), .73
Mother's education (Ref: College graduate/post graduate level)	0.53 (0.26-1.11), .09	0.51 (0.24-1.08), .08	1.47 (0.64-3.39), .36	1.43 (0.62-3.32), .40	0.73 (0.29-1.82), .50	0.72 (0.29-1.82), .49
Survivor has public or no health insurance (Ref: Private insurance)	1.07 (0.50-2.26), .87	0.94 (0.43-2.03), .87	0.44 (0.18-1.10), .08	0.41 (0.16-1.04), .06	0.69 (0.27-1.78), .44	0.66 (0.25-1.71), .39
Cardiovascular CHC [f] (Ref: None)	0.58 (0.29-1.16), .12	0.53 (0.26-1.08), .08	2.10 (0.92-4.79), .08	2.11 (0.92-4.84), .08	0.47 (0.19-1.15), .10	0.47 (0.19-1.15), .10
Endocrine CHC (Ref: None)	1.13 (0.56-2.28), .74	1.08 (0.52-2.25), .84	0.44 (0.19-1.03), .06	0.44 (0.19-1.02), .06	1.17 (0.48-2.84), .73	1.11 (0.45-2.72), .82
Hematologic CHC (Ref: None)	2.42 (1.01-5.77), .05	2.46 (1.01-6.00), .05	1.54 (0.55-4.28), .41	1.52 (0.54-4.26), .42	1.88 (0.61-5.80), .27	1.89 (0.62-5.80), .26
Neurologic CHC (Ref: None)	2.60 (1.23-5.50), .01	2.91 (1.34-6.35), .01	2.01 (0.88-4.62), .10	2.05 (0.89-4.71), .09	5.90 (2.35-14.83), <.001*	6.04 (2.39-15.26), <.001*
Family conflict	1.04 (0.73-1.46), .84	0.97 (0.68-1.40), .88	1.25 (0.86-1.81), .25	1.24 (0.85-1.81), .26	0.89 (0.56-1.41), .61	0.86 (0.54-1.39), .54
High area deprivation (Ref: ≤90th percentile SVI [g])	1.11 (0.31-3.94), .87	1.31 (0.36-4.71), .68	0.28 (0.03-2.56), .26	0.31 (0.04-2.74), .29	4.86 (1.32-17.82), .02	5.22 (1.39-19.54), .01
Caregiver anxiety	1.29 (0.91-1.83), .16	1.22 (0.85-1.76), .28	1.18 (0.79-1.76), .42	1.15 (0.76-1.72), .51	0.89 (0.57-1.38), .59	0.87 (0.56-1.35), .53
Survivor's meaning/purpose	---	0.52 (0.36-0.75), <.001*	---	0.80 (0.54-1.18), .25	---	0.75 (0.48-1.18), .22
**Moderate/high symptom burden (Ref: No symptom burden)**						
Diagnosis (Ref: solid tumors)						
CNS tumors	0.37 (0.04-3.58), .39	0.32 (0.03-3.49), .35	4.99 (0.16-160.47), .36	5.07 (0.14-188.27), .38	0.27 (0.02-3.77), .33	0.31 (0.02-4.54), .39
Hematologic cancer	1.90 (0.54-6.71), .32	1.60 (0.44-5.86), .48	11.63 (0.80-169.40), .07	10.71 (0.75-152.32), .08	1.14 (0.20-6.47), .88	0.89 (0.13-5.93), .91
Time since diagnosis	0.63 (0.16-2.48), .50	0.72 (0.17-3.00), .65	0.72 (0.07-7.21), .78	0.92 (0.08-9.99), .94	1.84 (0.24-13.94), .56	2.83 (0.32-25.25), .35
Female sex (Ref: Male)	4.48 (1.15-17.54), .03	5.04 (1.21-20.96), .03	4.28 (0.38-47.94), .24	5.15 (0.42-63.97), .20	1.45 (0.28-7.62), .66	1.20 (0.19-7.61), .84
Age	1.65 (0.41-6.57), .48	1.44 (0.34-6.11), .62	1.19 (0.11-12.84), .89	0.95 (0.08-11.42), .97	0.50 (0.07-3.77), .50	0.32 (0.04-2.90), .31

© 2024 Horan MR et al. *JAMA Network Open.*

Determinants	Feel sick to your stomach (nausea)		Problems with not being able to poop		Numbness or tingly feeling in your hands or feet	
	Model 1 [b]	Model 2 [c]	Model 1	Model 2	Model 1	Model 2
	RR [d] (95% CI), p	RR (95% CI), p	RR (95% CI), p	RR (95% CI), p	RR (95% CI), p	RR (95% CI), p
**Moderate/high symptom burden (Ref: No symptom burden)**						
Mother's education (Ref: College graduate/post graduate level)	0.99 (0.27-3.61), .99	0.89 (0.22-3.53), .87	N/A	N/A	0.27 (0.04-1.78), .17	0.28 (0.04-2.20), .23
Survivor has public or no health insurance (Ref: Private insurance)	0.48 (0.11-2.12), .33	0.35 (0.07-1.77), .21	N/A	N/A	1.63 (0.28-9.65), .59	1.15 (0.18-7.42), .88
Cardiovascular CHC (Ref: None)	0.57 (0.17-1.91), .37	0.57 (0.16-1.97), .37	0.30 (0.03-3.01), .30	0.35 (0.03-3.61), .38	2.16 (0.38-12.46), .39	1.69 (0.28-10.26), .57
Endocrine CHC (Ref: None)	1.00 (0.29-3.43), 1.00	1.02 (0.28-3.69), .98	2.15 (0.22-21.16), .51	1.55 (0.14-16.92), .72	1.41 (0.29-6.79), .67	1.56 (0.29-8.56), .61
Hematologic CHC (Ref: None)	1.23 (0.22-6.81), .81	1.21 (0.21-6.97), .83	N/A	N/A	5.04 (0.83-30.65), .08	7.50 (1.18-47.43), .03
Neurologic CHC (Ref: None)	4.39 (1.22-15.77), .02	4.59 (1.22-17.23), .02	5.50 (0.50-61.19), .17	4.66 (0.39-56.45), .23	18.69 (2.96-118.11), .002*	33.23 (3.85-287.29), .001*
Family conflict	1.12 (0.61-2.07), .72	1.13 (0.59-2.14), .71	1.60 (0.55-4.63), .39	1.65 (0.56-4.82), .36	1.24 (0.57-2.71), .58	1.18 (0.49-2.85), .71
High area deprivation (Ref: ≤90th percentile SVI)	N/A	N/A	N/A	N/A	10.46 (1.26-86.60), .03	14.19 (1.46-138.38), .02
Caregiver anxiety	0.71 (0.36-1.42), .33	0.62 (0.30-1.29), .20	0.32 (0.06-1.74), .19	0.30 (0.06-1.59), .16	0.92 (0.42-2.01), .83	0.97 (0.43-2.19), .93
Survivor's meaning/purpose	---	0.37 (0.19-0.75), .01	---	0.56 (0.15-2.02), .37	---	0.35 (0.15-0.83), .02

[a] Cancer treatment was not included because treatment is highly correlated with CHCs. Models with mouth pain as the outcome had convergence issues and are not reported here.
[b] Model 1 = personal characteristics and contextual variables
[c] Model 2 = personal characteristics, contextual variables, and personal meaning/purpose
[d] RR = risk ratio
[e] CNS = central nervous system
[f] CHC = chronic health condition
[g] SVI = Social Vulnerability Index
N/A = empty cell
* Significant when corrected for multiple comparisons using Bonferroni's correction (p = 0.004)

© 2024 Horan MR et al. *JAMA Network Open.*
