# Supplementary material for: Multilevel Characteristics of Cumulative Symptom Burden in Young Survivors of Childhood Cancer
Source: JAMA Netw Open. 2024 May 7;7(5):e2410145. doi: 10.1001/jamanetworkopen.2024.10145 (PMC11077392; doi:10.1001/jamanetworkopen.2024.10145)
Supplement: Supplement 2. — Data Sharing Statement [file jamanetwopen-e2410145-s002.pdf]

Data Sharing Statement

Horan. Multilevel Characteristics of Cumulative Symptom Burden in Young Survivors of Childhood Cancer. *JAMA Netw Open*. Published May 07, 2024.
doi:10.1001/jamanetworkopen.2024.10145

Data
Data available: Yes
Data types: Deidentified participant data, Data dictionary
How to access data: i-chan.huang@stjude.org
When available: With publication

Supporting Documents
Document types: None

Additional Information
Who can access the data: i-chan.huang@stjude.org
Types of analyses: For any purpose
Mechanisms of data availability: with investigator support
